# Panthenol Additives with Multiple Coordination Sites Induce Uniform Zinc Deposition and Inhibited Side Reactions for High Performance Aqueous Zinc Metal Battery

**DOI:** 10.1002/advs.202402074

**Published:** 2024-07-21

**Authors:** Ping Luo, Gongtao Yu, Wenwei Zhang, Zhen Huang, Yipeng Wang, Dongyao Zhu, Feiyang Chao, Yuyua Wang, Wenhui Zhong, Zhaoyang Wang, Shijie Dong, Qinyou An

**Affiliations:** ^1^ Hubei Engineering Laboratory of Automotive Lightweight Materials and Processing Hubei Provincial Key Laboratory of Green Materials for Light Industry School of Materials and Chemical Engineering Hubei University of Technology Wuhan 430068 P. R. China; ^2^ Hubei Longzhong Laboratory Xiang Yang Hubei 441000 P. R. China; ^3^ State Key Laboratory of Advanced Technology for Materials Synthesis and Processing Wuhan University of Technology Wuhan 430070 P. R. China; ^4^ School of Chemistry and Materials Science Hubei Engineering University XiaoGan 432000 P. R. China

**Keywords:** anode‐electrolyte interface, hydrogen bonding network, panthenol, solvation structure, zinc metal batteries

## Abstract

Application of aqueous zinc metal batteries (AZMBs) in large‐scale new energy systems (NESs) is challenging owing to the growth of dendrites and frequent side reactions. Here, this study proposes the use of Panthenol (PB) as an electrolyte additive in AZMBs to achieve highly reversible zinc plating/stripping processes and suppressed side reactions. The PB structure is rich in polar groups, which led to the formation of a strong hydrogen bonding network of PB−H_2_O, while the PB molecule also builds a multi‐coordination solvated structure, which inhibits water activity and reduces side reactions. Simultaneously, PB and OTF^−^ decomposition, in situ formation of SEI layer with stable organic‐inorganic hybrid ZnF_2_‐ZnS interphase on Zn anode electrode, can inhibit water penetration into Zn and homogenize the Zn^2+^ plating. The effect of the thickness of the SEI layer on the deposition of Zn ions in the battery is also investigated. Hence, this comprehensive regulation strategy contributes to a long cycle life of 2300 h for Zn//Zn cells assembled with electrolytes containing PB additives. And the assembled Zn//NH_4_V_4_O_10_ pouch cells with homemade modules exhibit stable cycling performance and high capacity retention. Therefore, the proposed electrolyte modification strategy provides new ideas for AZMBs and other metal batteries.

## Graphical abstract

In this paper, by adding the panthenol additive to the electrolyte, a strong hydrogen‐bonding network is formed, and a multi‐coordinated dissolution structure is constructed to reduce the side reactions. Simultaneously, panthenol and OTF^−^ decomposition, in situ formation of SEI layer with stable organic‐inorganic hybrid ZnF_2_‐ZnS interphase on Zn anode electrode, can inhibit water penetration into Zn and homogenize the Zn^2+^ plating.

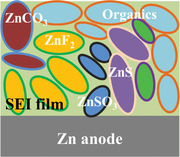

## 1 Introduction

Optimization of energy systems and the transformation of the new energy systems (NESs) are necessary to achieve energy conservation, emission reduction, and carbon neutrality initiatives.^[^ ^1^ ^]^ Lithium‐ion organic batteries exhibit low safety and high cost; therefore, aqueous zinc metal batteries (AZMBs) emerge as one of the most promising candidates for next‐generation conventional NESs owing to their low cost, high ionic conductivity, and high safety.^[^ ^2^ ^]^ In contrast to other metal anodes, zinc metal is an ideal anode candidate for aqueous metal batteries.^[^ ^3^ ^]^ Owing to its moderate redox potential (−0.762 V versus standard hydrogen electrode), high theoretical specific capacity (820 mA h g^−1^/5854 mA h cm^−3^), abundant reserves, and a large amount of Zn^2+^ insertion/extraction facilitated by the host material acting as a cathode.^[^ ^2^, ^4^ ^]^

However, the thermodynamic instability of zinc metal in aqueous environments during the electroplating and stripping processes leads to inhomogeneous Zn^2+^ diffusion, which induces an uneven distribution of the electric field.^[^ ^5^ ^]^ The uneven Zn deposition leads to an elevated surrounding pH, thus resulting in severe parasitic reactions, corrosion/passivation, and zinc dendrite growth on the zinc metal anode.^[^ ^2d^ ^]^ Hence, the zinc anode instability could be attributed to the following two aspects: first, Zn^2+^ ions are coordinated by a large number of free H_2_O molecules in the mildly neutral aqueous electrolyte.^[^ ^6^ ^]^ These coordinated H_2_O molecules have weak hydroxide bonds and exhibit high activity during electroplating, leading to de‐protonation of H_2_O, which promotes H_2_O decomposition, thus resulting in severe parasitic reactions.^[^ ^7^ ^]^ Second, there are a large number of H_2_O−H_2_O bonds, resulting in (H−O−H) hydrogen bonding networks in pure water‐based electrolytes,^[^ ^8^ ^]^ which remarkably affect the zinc precipitation‐dissolution process, primarily during the ultrafast diffusion of protons and hydroxides and ionic product separation,^[^ ^9^ ^]^ thus, accelerating the water decomposition and related parasitic reactions. Consequently, dendrites and “dead Zn” are formed, further affecting the compound efficiency (CE) of Zn^2+^ precipitation/exfoliation and the cell lifetime.^[^ ^10^ ^]^ Therefore, reducing the coordination of Zn^2+^ with free water and disrupting the hydrogen‐bonding network between H_2_O molecules to reduce the water activity in the aqueous electrolyte is one of the most effective means for improving the anode stability and inhibiting the side reactions.

The current research efforts are focused on inhibiting water activity and breaking the hydrogen‐bonding network by optimizing the solvated structure. Using aqueous electrolytes with a high salt content, eutectic water, and deep eutectic solvent methods, reduces the bonding strength between water molecules.^[^ ^11^ ^]^ For instance, Shi et al.^[^ ^12^ ^]^ reported a new aqueous deep eutectic solvent with acetamide, ZnCl_2_, and H_2_O as aqueous electrolytes for AZMBs, which improved the Zn anode stability. However, the inherent high viscosity and low ionic conductivity of these systems limited their potential applicability in aqueous batteries.^[^ ^13^ ^]^ Hence, finding suitable additives to address these issues remains challenging. Among them, the panthenol molecule is widely used in medicine whitening, it is a low‐cost, environmentally friendly, non‐toxic, harmless, and other characteristics of the compound. At the same time, panthenol is a long‐chain compound with multiple polar groups that can effectively improve the electrolyte environment and enhance the stability of zinc anode electrode to achieve excellent performance of zinc metal batteries.

Therefore, in this paper, the effect of PB in the electrolyte is investigated by adding trace amounts of PB (PB‐*x*, where *x* is the molar mass ratio of H_2_O to PB) and 2 m Zn(OTF)_2_. Experimental and theoretical calculations showed that the addition of PB not only optimizes the electrolyte environment of AZMBs and suppressed the generation of side reactions, but also constructs a multi‐coordinated solvation structure, improves the coulombic efficiency of Zn^2+^ deposition, and therefore achieves a highly stable and reversible electrochemical cycle. Therefore, this study provides new insights into the utilization of electrolyte additives for successfully constructing AZMBs.

## 2 Results and Discussion

### 2.1 Reconstruction of Solvated Structure and the Hydrogen Bonding Network

**Figure 1:**
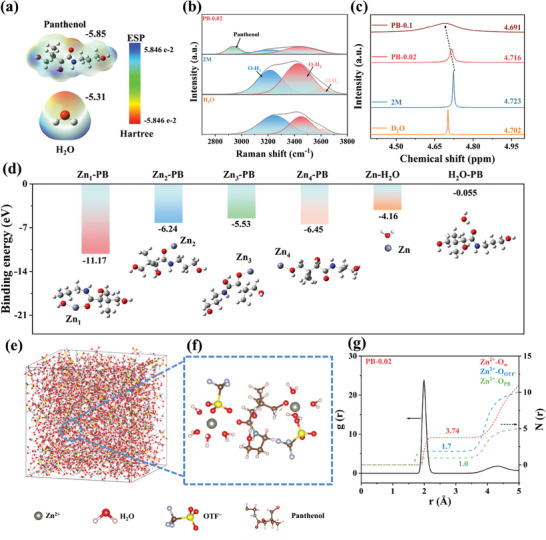
Reconstruction of hydrogen bonding network and solvated structure. a) Electrostatic potential mapping of PB (top) and H_2_O (bottom). b) Raman spectra of H_2_O, 2 m, and PB‐0.02 electrolytes. c) ^1^H NMR spectra of pure D_2_O and various PB‐containing electrolytes. d) Interaction behaviors among H_2_O, PB, and Zn^2+^ and their corresponding binding energies. e) 3D snapshot of MD simulations for PB‐0.02 electrolyte and f) partial enlarged snapshot representing Zn^2+^ solvation structure. g) Simulated radial distribution functions (RDFs) for Zn^2+^−O_W_, Zn^2+^−O_OTF_^−^, and Zn^2+^−O_PB_ collected from MD simulations in PB‐0.02 electrolyte.

The effect of PB additives on the hydrogen bonding network and solvated structure of Zn^2+^ has been investigated via DFT calculations, MD simulations, as well as via Fourier transform infrared (FTIR) and Raman spectroscopies. The electrostatic potential calculations of PB and H_2_O (**Figure** **1**a) reveal that PB has a greater electrostatic potential energy than that of H_2_O, and its C−N and hydroxyl groups show a greater charge‐distribution and attraction toward hydrogen atoms as compared to that of O−H, suggesting that PB can reconfigure the hydrogen‐bonding network, thus potentially affecting the remodeling of solvated sheaths.^[^ ^10a^ ^]^ As depicted in Figure S1 (Supporting Information), the wave number of the O−H stretching vibration in the electrolyte gradually increases, whereas the intensity weakens and the peak broadens with the increasing PB quantity in the 2800–3800 cm^−1^ Raman spectral range.^[^ ^14^ ^]^ The higher wave number indicates greater bonding energy of O−H in water owing to a decrease in the number of hydrogen bonds and disruption of the hydrogen bonding network. In addition to the peak shift, the tensile and bending vibrations of O−H are considerably reduced owing to a decrease in water content.^[^ ^15^ ^]^ Further, the Raman peaks of the electrolyte were fitted, and the peaks were classified into strong hydrogen bonding (O−H_1_) generated by the coupling of water molecules, weaker hydrogen bonding (O−H_2_), and non‐hydrogen bonding water molecule (O−H_3_) (Figure 1b; Figure S2a, Supporting Information).^[^ ^10c^ ^]^ The distribution of the hydrogen bonding types for different electrolytes was determined by integrating the respective peak areas, as shown in Figure S2b (Supporting Information). Compared to that of pure water, the peak area corresponding to O−H_1_ decreases substantially with the addition of zinc salt, indicating the incorporation of water molecules in the solvated structure. In contrast, the O−H_1_ area gradually increases with the addition of the PB additive because PB has a remodeling effect on the solvated sheath of hydrated Zn^2+^ and releases more coordinated water molecules into the electrolyte system. The proportions of O−H_2_ and O−H_3_ components increase in the 2 m electrolyte compared to that in pure water, indicating that water molecules and OTF^−^ ions in the vicinity of Zn^2+^ are involved in hydrogen bonding interactions. When PB is introduced into the electrolyte, the proportion of O−H_2_ gradually increases, indicating remodeling of the hydrogen bonding network in the electrode solution. This reconfiguration could be attributed to the bonding between the hydroxyl group of PB acting as an electron donor and the hydrogen atoms in water, thus constructing a stronger hydrogen‐bonding network. Consequently, the previously active water molecules are not “free,” and a large number of hydrogen bonding networks are created between PB and water molecules in the electrolyte.

Additionally, the FTIR spectroscopy demonstrates the effect of PB on the hydrogen bonding network reconfiguration. The peaks (Figure S3, Supporting Information) at 3220, 3420, and 3603 cm^−1^ could be attributed to three different water molecule environments: bulk water, cluster PB−H_2_O, and isolated PB−H_2_O, respectively (Figure S4a, Supporting Information).^[^ ^16^ ^]^ The introduction of PB considerably suppresses the O−H stretching vibrations in the 2700–4000 cm^−1^ range, indicating that the hydrogen bonding network of bulk water is severely disrupted. In contrast, the increase in the ratio of cluster PB−H_2_O to isolated PB−H_2_O (Figure S4b, Supporting Information) confirms the formation of a new type of PB−H_2_O hydrogen bonding. Additionally, the bending vibration of water in the 1600–1700 cm^−1^ range (Figure S5, Supporting Information) exhibits a slight blue shift, supported by the enhanced hydrogen bonding between PB and water molecules. Hence, PB imposes effective limitations on free water molecules, thus reducing hydrogen evolution reaction (HER) and water‐induced corrosion.^[^ ^17^ ^]^

Nuclear magnetic resonance (NMR) was performed to reveal the overall effect of PB on the Zn^2+^ solvated structure. Figure 1c exhibits a ^1^H chemical shift at 4.702 ppm in the pure D_2_O sample, whereas the D_2_O peak in the 2 m electrolyte shifts to 4.723 ppm, indicating a stronger interaction between Zn^2+^ and water. As the PB additive content increases, the ^1^H peak shifts from 4.716 to 4.691 ppm, which could be attributed to the introduction of PB additives that release water with multiple coordination sites in the solvated structure, thus weakening the interaction between Zn^2+^ and water molecules. Further, the binding energies and interaction behaviors of Zn^2+^ with PB and water were investigated (Figure 1d). The results reveal that the binding energies of PB molecules with Zn^2+^ are higher than those with Zn^2+^ and water at different sites, thus confirming that the PB molecule addition affects the Zn^2+^ solvated structure. Moreover, the PB molecule has a longer molecular chain and a greater number of coordination sites with Zn^2+^, resulting in the construction of its multi‐coordinated solvated structure with Zn^2+^. Subsequently, MD simulations were performed to analyze the solvated sheath structures of the PB‐0.02 and 2 m electrolyte systems. The calculations reveal that the solvated structure of Zn^2+^ in the presence of pure Zn(OTF)_2_ comprises 5.05 water molecules and 1.4 OTF^−^ (Figure S6a,b, Supporting Information). When PB is introduced into the electrolyte (Figure 1e,f), a novel multi‐coordinated solvated structure is constructed with at least one water molecule replaced by the hydroxyl group of PB. Based on the calculation results for the different electrolyte models, the corresponding radial distribution functions (RDFs) of the molecules and their coordination numbers were derived. Compared with the pure electrolyte (Figure S6c, Supporting Information), the average coordination number of water in PB‐0.02 is 3.74 (Figure 1g), indicating the reduction of active water molecules around Zn^2+^ ions. The reduction of allotropic water effectively inhibits aqueous corrosion on the anode surface.

Additionally, the average coordination numbers of Zn^2+^−O_PB_ and Zn^2+^−O_OTF_^−^ in the solvated shell layer are 1.00 and 1.70, respectively, indicating that both PB and OTF^−^ actively participate in the coordination of solvated structure with Zn^2+^. Moreover, the coordination number of Zn^2+^−O_H2O_ decreases, and that of Zn^2+^−O_PB_ increases, further confirming the construction of multi‐coordination solvated structures. Therefore, these structures improve the efficiency of PB participation, reduce cost, and inhibit the HER as well as side reactions.^[^ ^18^ ^]^ At the same time, Figure S7 (Supporting Information) shows the conductivity versus PH test of electrolyte with different content of PB molecules. It can be seen in the figure that the electrolyte conductivity decreases rapidly as the H_2_O:PB ratio exceeds 0.02. This is mainly due to the fact that as the content of PB molecules increases, the macromolecular groups in the solvated structure are further increased, resulting in the inability of ions to move rapidly in the electrolyte. Meanwhile, with the elevated PB added in the electrolyte, the pH increased from 3.3 to 5.0, indicating that with the PB molecules can inhibit the activity of water, which effectively reduces the number of free H^+^ ions and prevents the negative electrode corrosion.^[^ ^2c^ ^]^

### 2.2 Effect of Multi‐Coordination Solvated Structures on Zn^2+^ Deposition and Preferential Adsorption of PB

**Figure 2:**
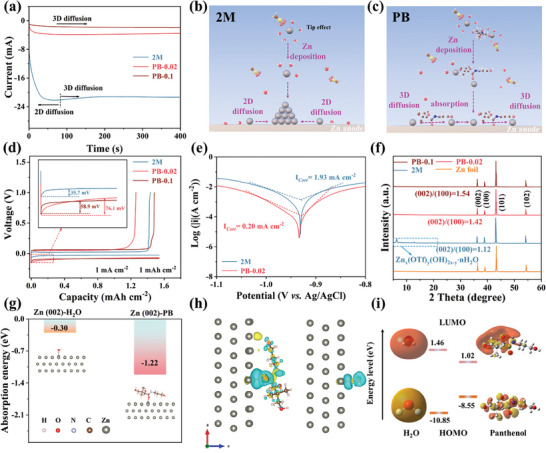
Effect of solvation structure on Zn^2+^ deposition and preferential PB adsorption. a) CA results of Zn//Zn symmetrical cells using electrolyte with and without PB. b,c) Schematics of the Zn^2+^ diffusion and deposition on Zn anode. d) Typical voltage‐time curves of Zn//Cu asymmetric cells in 2 m, PB‐0.02 and PB‐0.1 electrolytes. e) Tafel plots of Zn//Zn symmetric cells assessed at 2 mV s^−1^ scan rate in 2 m and PB‐0.02 electrolytes. f) Corresponding XRD patterns of Zn plates in different electrolytes. g) Comparison of adsorption energies of H_2_O and PB molecules on the (002) plane of Zn anodes. h) Charge density differences (yellow: the accumulation of electrons; blue: the depletion of electrons) between PB (left) and H_2_O (right) molecules along the plane of the corresponding isosurface (002). i) LUMO and HOMO isosurfaces of H_2_O (left) and PB (right) molecules.

The effect of the multi‐coordination solvated structures on Zn^2+^ deposition was explored using electrochemical tests. The influence of PB additives on the Zn^2+^ deposition kinetics was verified using chronoamperometry (CA) testing.^[^ ^19^ ^]^ As shown in **Figure** **2**a, the current density in the 2 m pure electrolyte continuously increases above 80 s, indicating random 2D diffusion at the electrode surface. Further, Zn^2+^ ions diffuse within the 2D plane to identify highly active deposition sites and accumulate at localized tips, leading to dendrite formation (Figure 2b).^[^ ^20^ ^]^ In contrast, the anode in the PB‐0.02 electrolyte exhibits shorter 2D diffusion (within 20 s) and stable spatial 3D diffusion compared to those of PB additives, reflecting the constraining effect of adsorbed PB molecules on Zn^2+^ diffusion.^[^ ^21^ ^]^ The PB molecules preferentially adsorb on the zinc anode, thus enhancing the spatial effect, promoting homogeneous Zn^2+^ deposition, and inhibiting dendrite growth (Figure 2c). The multi‐coordinated solvated structure promotes the preferential detachment of water molecules during the deposition and dissolution processes and inhibits the side reactions.

The voltage distribution of a Zn//Cu asymmetric cell assembled with each of the three electrolytes at a current density of 1 mA cm^−2^ is shown in Figure 2d. The nucleation over‐potential in the 2 m electrolyte is 35.7 mV, whereas in the PB‐0.2 electrolyte, it increases to 76.1 mV. Therefore, the nucleation over‐potential increases with increasing PB content (Figure S8, Supporting Information).^[^ ^22^ ^]^ Moreover, the multi‐coordination solvation structure constructed by PB reduces the number of Zn^2+^ nucleation sites. It decreases the rate of Zn^2+^ deposition, which inhibits the growth of dendrites, thus leading to better crystal orientation, as demonstrated by the CV curves of 2 m, PB‐0.02, and PB‐0.1 electrolytes in the Zn//Cu asymmetric cells (Figure S9a–c, Supporting Information). The increase in the nucleation potential confirms the modulation of Zn^2+^ nucleation by the multi‐coordination solvation structure. Linear scanning voltammetry (LSV) measurements of the Zn//Cu asymmetric cell in Figure S10 (Supporting Information) show that the potential for hydrogen precipitation increases with a gradual increase of PB concentration in the Zn(OTF)_2_ electrolyte. This increase indicates a reduced potential for water decomposition, thus supporting the reduction in the number of active water molecules at the interface. Consequently, its electrochemical window expands.^[^ ^23^ ^]^

Tafel plots of Zn//Zn symmetric cells using different electrolytes are shown in Figure 1e and Figure S11 (Supporting Information) to assess the corrosion behavior of the zinc anode. The corrosion current density of PB‐0.02 is substantially higher than that of the pure electrolyte.^[^ ^24^ ^]^ With increased PB content, the corrosion current density decreases, confirming the inhibiting effect of PB addition on the corrosion reflection. The electrochemical impedance spectroscopy (EIS) impedance diagram (Figure S12, Supporting Information) reveals that the proportion of water decreases when the additive PB content increases, and the solvation structure becomes larger, which enhances the zinc deposition energy, leading to difficulties in mass transfer, thus increasing the impedance value. Figure S13 (Supporting Information) shows that metal Zn has a high contact angle (84.23°) in 2 m pure electrolyte, whereas PB‐0.02 (47.14°) and PB‐0.1 (76.38°) have lower contact angles. These results confirm the wettability of zinc metal by PB molecules. Therefore, selecting the electrolyte with the appropriate H_2_O:PB ratio is necessary, and PB‐0.02 is the most suitable for a comprehensive comparison.

To explore the influence of added PB on the anode‐electrolyte interface, it was characterized by XRD after 50 cycles at a current density of 1 mA cm^−2^ and an area capacity of 1 mA h cm^−2^. Since the surface energy of the six‐sided arrangement of the Zn (002) crystal plane is lower than that of the Zn (100) plane, the uniform deposition of Zn depends on (002)/(100).^[^ ^2^, ^10^, ^25^ ^]^ As shown in Figure 2f, a large amount of by‐products Zn_x_(OTF)_y_(OH)_2x‐y_·nH_2_O was produced at the anode‐electrolyte interface in the 2 m pure electrolyte, and the (002)/(100) peak intensity of the Zn anode was much lower than that of the pure Zn sheet, which indicated that Zn^2+^ in the 2 m electrolyte was mainly deposited from the (100) peak surface during the deposition process. In contrast, the by‐products at the anode interface gradually decreased with the increase of PB additive (Figure S14, Supporting Information), and the (002)/(100) peak increased from 1.12 (PB‐0.005) to 1.54 (PB‐0.1), which further verified that the additive PB could enhance the Zn deposition along the (002) plane and promote the smooth and dendrite‐free deposition at the anode‐electrolyte interface.^[^ ^26^ ^]^

The XRD results were further verified via XPS characterization of the Zn surface after cycling. Figure S15a (Supporting Information) reveals a marginal increase in the Zn 2p binding energy with increasing PB content, primarily attributed to the composition of the solvated structure with multiple coordination sites in the PB electrode solution and Zn deposition on the 002 surface. Further, N 1s (Figure S15b, Supporting Information) detected on the Zn surface implies that PB is also involved in Zn^2+^ deposition and dissolution. Additionally, a decrease in the S 2p intensity on the Zn surface demonstrates the inhibition of byproduct production by PB (Figure S15c, Supporting Information). The electrolyte‐containing PB additive enhances the Zn deposition along the (002) plane, which could be primarily attributed to PB adsorption on the Zn anode. The adsorption energies of PB on Zn (100) and (002) crystal faces were determined via DFT calculations, as shown in Figure 2g and Figure S16 (Supporting Information), respectively. The adsorption energy of PB at Zn (100) (−1.67 eV) is lower than that at the (002) crystal face (−1.22 eV), indicating that PB preferentially adsorbs at the Zn (100) crystal plane. Therefore, the deposition of Zn^2+^ ions is hindered at the (100) crystal plane and induced on the (002) crystal plane. The adsorption energies of PB on both crystal surfaces (−1.22/−1.67 eV) are lower than that of water (−0.30/−0.23 eV), enabling the preferential adsorption of PB on the Zn anode, thus resulting in a water‐poor zone at the anode‐metal interface, which inhibits the direct contact between Zn metal and water molecules and hinders the side reactions. Charge‐density difference calculations were performed to determine whether the PB absorption was physical or chemical.^[^ ^27^ ^]^ Figure 2h depicts the overlapping of the electron clouds between the hydroxyl groups of Zn and PB during the adsorption process, and the electrons transfer from the Zn atoms to the PB molecules, thus resulting in chemisorption. The N 1s (Figure S15b, Supporting Information) was detected at the anode interface using XPS after 50 cycles, further demonstrating the chemisorption of PB at the anode interface. Comparing the charge‐density difference of water in the pure electrolyte to that of PB reveals that the consumption and accumulation of charge on the PB molecules is marginally higher as the highest occupied molecular orbital (HOMO) energy level of the PB (−8.55 eV) is larger than that of the water molecule (−10.85 eV) (Figure 2i). Hence, the stronger affinity between the anode interface and PB is more prone to charge transfer.

### 2.3 PB and OTF^−^ Co‐Derived SEI Film and Stable Anodic Interfaces

**Figure 3:**
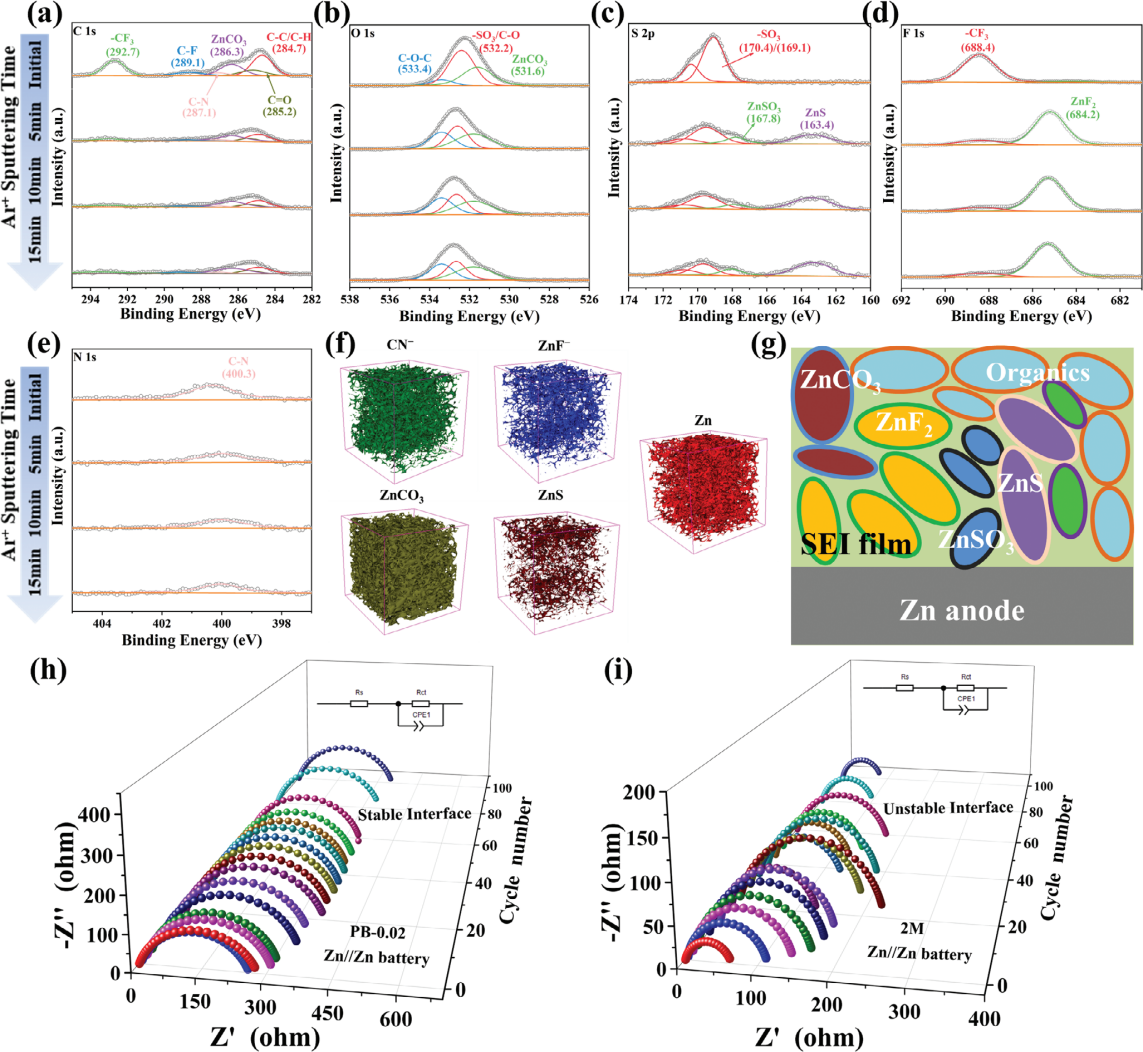
Time profiles of a) C 1s, b) O 1s, c) S 2p, d) F 1s, and e) N 1s spectra generated after Ar^+^ sputtering for 0, 5, 10, and 15 min. f) ToF‐SIMS 3D rendering model of CN^−^, ZnF^−^, ZnCO_3_, ZnS, and Zn after 300s etching. g) Schematic illustration of the interphase chemistry on Zn electrode (Zinc anode after 50 cycles of PB‐0.02 electrolyte). *In‐situ* EIS curves of the Zn//Zn battery with h) 2 m, and i) PB‐0.02.

Based on the HUMO/LUMO values of PB molecules and water, it was shown that the LUMO level of PB molecules (Figure 2i) is lower than that of water, indicating that the PB molecules are preferentially reduced and decomposed.^[^ ^28^ ^]^ The decomposition of the PB molecules out of the zinc‐anode interface was further verified by XPS etch, TOF‐SIMS, and other testing methods. The results show that the decomposition of the PB molecules occurs on the anode surface to form SEI membranes with organic‐inorganic hybridization rich in ZnF_2_‐ZnS‐ZnCO_3_ bonding phases. XPS etching analysis proved that PB molecules generate decomposition, comparing the C 1s spectra of 2 m (Figure S17, Supporting Information) and PB‐0.02 (**Figure** **3**a) zinc anode electrode, it was found that the peaks of ZnCO_3_, C─N, and C═O could still be detected in the anode electrode of PB‐0.02 after etching for 10 µm, whereas the peaks of the C 1s in the anode electrode of 2 m were basically disappeared after etching for 10 µm, which is in accordance with the results of TOF‐SIMS, and it proved that the PB molecules with OTF^−^ generated SEI layer.^[^ ^7b^ ^]^ Comparing the O 1s (Figure 3b) spectra of the two, it was found that a large number of ZnO/Zn(OH)_2_ peaks were generated in 2 m, which was mainly due to the side reactions such as corrosion and passivation at the anode interface.

On the other hand, no relevant reaction peaks were observed in PB‐0.02, which proves that the SEI layer formed by the decomposition of PB molecules can effectively inhibit the generation of by‐products. The composition of the SEI film was further analyzed by comparing the XPS of the S 2p (Figure 3c), F 1s (Figure 3d), and N 1s (Figure 3e) spectra, and it was found that both ZnF_2_, ZnSO_3_ and ZnS generated in PB‐0.02 were below 10 um, and the intensity of the C─N peaks of PB‐0.02 gradually weakened with the increase of etching depth.^[^ ^24a^ ^]^ For the F 1s and S 2p spectra, the SEI surface mainly involves organic ─CF_3_ and ─SO_3_ components, which come from the incomplete reduction of OTF^−^ anion or trace salt residue on the Zn electrode. These results indicate that PB and OTF^−^ co‐derived SEI mainly form an organic‐rich outer layer and an inorganic ZnF_2_‐ZnS inner layer, which can protect the Zn surface from water and guide the diffusion of Zn^2+^ (Figure 3g). The presence of C─N^−^ was observed in the TOF‐SIMS 3D rendering model by etching the anode electrode interface for 300 s. The substances ZnS, ZnF_2_, and ZnCO_3_ were also detected. Whereas in the pure electrolyte there is no detection with C─N^−^, as well as comparing PB‐0.02 (Figure 3f), 2m (Figure S18, Supporting Information) has only a small amount of ZnF_2_ and ZnCO_3_, which further indicates that the PB molecule produces decomposition at the anode electrode interface.^[^ ^29^ ^]^ These produced ZnS, ZnF_2_, and ZnCO_3_ with high corrosion resistance and zinc ionic conductivity, while the SEI film formed by the decomposition of OTF^−^ and panthenol molecules protects the zinc surface from water, guides the diffusion of Zn^2+^, and improves the stability of the zinc‐anode interface.

The thickness of the SEI layer is also a mechanism that affects the performance of the battery. From the XPS etching in Figure S19a–e (Supporting Information), it can be seen that the intensity of the C 1s peak in the XPS spectral peak of PB‐0.1 is much higher than that of PB‐0.02 (Figure 3a–e) and 2 m (Figure S17a–e, Supporting Information). This suggests that as the PB content in the electrolyte rises, more PB molecules produce decomposition at the negative electrode interface during the first few turns from discharge, thickening the SEI layer at the negative electrode interface, which is further confirmed by the XPS spectra of its N 1s. When the SEI layer gradually becomes thicker, its zinc ion nucleation over‐potential at the anode interface increases dramatically (Figure S9, Supporting Information), resulting in the zinc ions not being able to nucleate smoothly, which in turn inhibits the electrochemical performance of the battery. Meanwhile, the thickness of the SEI is greatly increased, it results in the zinc ions not being able to be stripping/plating quickly enough during the discharge from thus causing change in the battery performance. Changes in the spectra of O 1s and F 1s were also observed in the PB‐0.1 electrolyte, where a significant decrease in the intensity of ZnCO_3_ and ZnF_2_ and an increase in the binding energy were observed, which are further evidence that the thickness of the SEI layer affects the deposition of zinc ions. The in situ EIS tests of the symmetrical Zn//Zn battery were further performed to investigate SEI layer at the interfacial electrochemical stability (Figure 3h,i). With the PB‐0.02 electrolyte, the interfacial transfer resistance (Rct) can be rapidly stabilized in a few cycles due to the uniform deposition of Zn^2+^ and the formation of a homogeneous and stable SEI layer. On the contrary, during the plating process, the Rct continued to decrease after 20 turns due to the inhomogeneous deposition of zinc ions in 2 m and the continuous decomposition of OTF^−^ groups at the anode interface, leading to the deterioration of the zinc/electrolyte interface. Therefore, PB‐0.02 electrolyte forms a stable SEI at the interface of zinc flake, which plays a key role in stabilizing the uniform deposition of zinc ions to inhibit the side reactions.

### 2.4 PB Additive Facilitates Uniform Zn^2+^ Deposition

**Figure 4:**
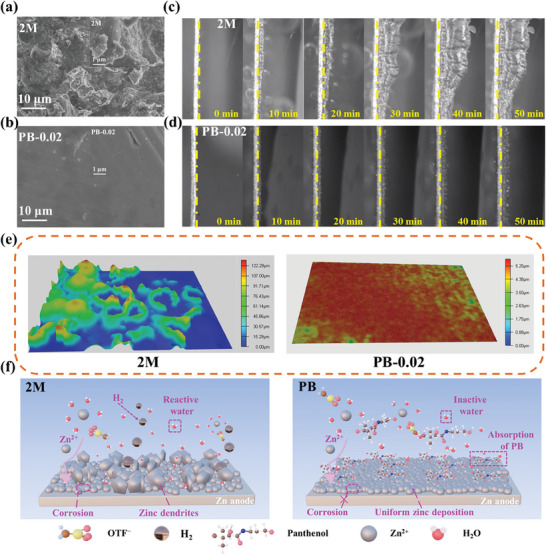
Optimization of PB additives for zinc deposition after dissolution. SEM images of zinc anodes stripped from zinc//zinc symmetric cells after cycling at 1 mA cm^−2^ and 1 mA h cm^−2^ in a) 2 m and b) PB‐0.02 electrolytes. In *situ* optical microscopy images of Zn plating in c) 2 m and d) PB‐0.02 electrolytes at a current density of 10 mA cm^−2^. e) Laser confocal scanning microscopy images of the anode surface with different electrolytes after cycling at 1 mA cm^−2^. (f) Schematic of Zn deposition behaviors in 2 m and PB‐0.02 electrolytes.

The controlled dendrite evolution and uniform deposition at the anodic interface by PB additives during Zn^2+^ stripping/plating were investigated using scanning electron microscopy (SEM), *in‐situ* optical, and laser confocal scanning microscopies. The SEM image in **Figure** **4**a, after fifty cycles at a current density of 1 mA cm^−2^, shows that the Zn anode surface in the 2 m pure electrolyte is loosely deposited and filled with Zn dendrites. SEM image at a higher magnification (Figure S21a, Supporting Information) shows several scattered fine Zn grains, owing to the prolonged 2D diffusion of Zn^2+^ at the anode interface in the pure electrolyte, which leads to the formation of Zn dendrites. With an increase in the PB additive content, Zn^2+^ deposition changes from 2D to 3D, which optimizes the Zn^2+^ deposition and inhibits the formation of zinc dendrites, as shown in Figure 4b, Figures S20a and S22a–c (Supporting Information). SEM at higher magnification (Figures S21b,c and S22d‐f,e, Supporting Information) reveals the absence of coarse zinc grains, further proving PB additive optimization.

The in‐situ optical microscopy images in Figure 4c,d, and Figure S20b (Supporting Information) reveal the Zn deposition process. In the 2 m pure electrolyte, uneven nucleation sites and protrusions appear after 10 min of deposition, thus triggering the “tip effect” and generating a large number of bubbles, which results in severe HER and side reactions. After 40 min of deposition, large zinc dendrites are formed on the surface. However, the electrodes in the PB electrolyte maintain their flat and uniform surface characteristics during the plating. Dendrites and bubbles are not observed even after 50 min of deposition at 10 mA h cm^−2^, indicating that the PB additive facilitates uniform, dendrite‐free zinc deposition and inhibits the HER and side reactions via strong hydrogen bond formation of PB‐H_2_O, thus constructing multi‐ligand solvated structures and preferentially adsorbing PB. Laser confocal scanning microscopy and SEM of the zinc anode section further demonstrate that the PB additive has a positive effect on the uniform deposition of Zn at the anodic interface (Figure 4e; Figure S23 and S24, Supporting Information). Cycling at 1 mA cm^−2^ current density with an area capacity of 1 mA h cm^−2^ reveals substantial dendrite generation in the 3D confocal images of the 2 m electrolyte, whereas no dendrite formation in the electrolytes containing PB‐0.02 and PB‐0.1 is observed. At the cross‐section of the Zn flakes, dendrites are observed in the 2 m electrolyte, indicating inhomogeneous deposition. In contrast, in the electrolyte containing PB, the cross‐section is homogeneous, and the absence of dendrites suggests a positive optimization effect of the PB additive. Figure 4f shows the overall mechanism for improving the stability of zinc anode using the PB‐H_2_O hybrid electrolyte based on the above experimental and theoretical studies.

### 2.5 Highly Reversible and Stable Zn Anode

**Figure 5:**
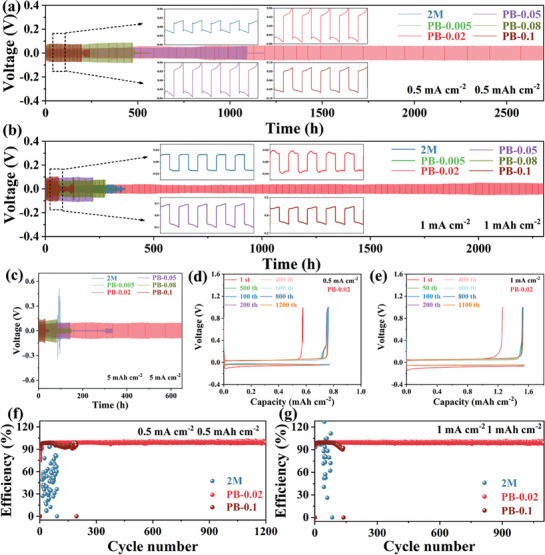
Highly reversible Zn plating/stripping behaviors. The performance of Zn//Zn battery with different electrolytes under a) 0.5 mA cm^−2^, 0.5 mA h cm^−2^, b) 1 mA cm^−2^, 1 mA h cm^−2^, and c) 5 mA cm^−2^, 5 mA h cm^−2^. d,e) Voltage profiles of Zn//Cu cells at different current densities. Effects of 2 m, PB‐0.02, and PB‐0.1 electrolytes on CE performances of the asymmetrical Zn//Cu battery at f) 0.5 mA cm^−2^ and g) 1 mA cm^−2^, respectively.

Different cell systems were used and electrochemical performance tests were conducted to determine the effects of stability and reversibility of PB additives on zinc metal anodes. Zn//Zn symmetric cells were evaluated at various current densities and area capacities to verify the optimization of the zinc metal anode stability by electrolyte‐containing PB additives. **Figure** **5**a,b reveal that PB‐0.02 achieves a remarkably stable cycle life of over 2300 h at 0.5 and 1 mA cm^−2^ current densities, respectively. However, the cycling performance of 2 m pure electrolyte at this current density does not exceed 200 h. Further, the voltage profiles of the electrolyte cells containing PB additives exhibit a considerably stable behavior compared to that of the 2 m pure electrode solution at 5 mA cm^−2^ and 5 mA h cm^−2^. The PB‐0.02 electrolyte cells display a stable plating/stripping process with a long‐term life of over 600 h. Therefore, the addition of the PB additive has a remarkable effect on the stability of the zinc anode compared to the 2 m pure electrolyte with a cycle life not exceeding 100 h.

The effects of electrolytes with various PB additive contents on the reversibility of the CE and Zn ions were investigated by reversible plating/stripping experiments of Zn//Cu cells at different current densities. Figure 5d,e confirm that the PB‐0.02 electrolyte exhibits stable voltage profiles for over 1100 cycles at current densities of 0.5 and 1 mA cm^−2^, respectively, the PB‐0.02 electrolyte exhibits a stable voltage profile for more than 1100 cycles. whereas at 5 mA cm^−2^ current density, stability of over 150 cycles is achieved (Figure S27c, Supporting Information). The 2 m electrode solution shows stable cycling for only 92 cycles owing to its dendritic deterioration and severe side reactions, and the best performance is demonstrated by PB‐0.02, compared to those by other ratios of PB additives (Figures S25–S27, Supporting Information). Figure 5f,g shows the CE of Zn//Cu asymmetric cells in different electrolytes, wherein the cells with the PB‐0.02 electrolyte achieve over 1100 stable cycles at 0.5 mA cm^−2^ and 0.5 mA h cm^−2^ as well as 1 mA cm^−2^ and 1 mA h cm^−2^ exhibiting an average CE of approximately 99.5%. However, dendritic growth and undesirable side reactions in the 2 m electrolyte lead to fluctuations in the CE of the cells after 50 cycles and battery failure after 90 cycles. Additionally, the cells with the PB‐0.02 electrolyte provide the best cycling stability at different current densities with an average CE exceeding 99.5% (Figure S28, Supporting Information). Thus the multi‐coordination solvated structure formed by the PB molecules improves the zinc deposition efficiency.

### 2.6 Evaluation of Zn‐Metal Full Cells

**Figure 6:**
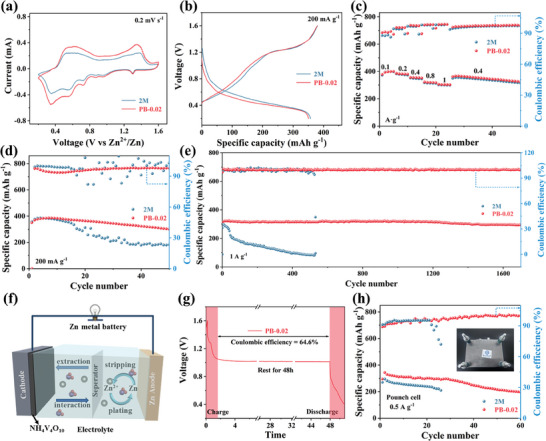
Performance of Zn//NHVO full cells. a) CV curves, b) Galvanostatic charge/discharge (GCD) curves, and c) Rate performance of Zn//NHVO full cells in different electrolyte solutions of 2 m and PB‐0.02. d) Cycling stability of the full cell at 200 mA g^−1^ current density. e) Long‐term cycling performance at 1 A g^−1^ current density. f) Schematic of zinc‐ion batteries (ZIBs) full cell device. g) Self‐discharge curves in PB‐0.02 electrolytes. h) Cycling stabilities of Zn//NHVO soft pack cells in PB‐0.02 and 2 m electrolytes.

To evaluate the practical applicability of the integrated electrolyte modulation strategy in battery systems, NH_4_V_4_O_10_ (NHVO) was selected as the cathode material to assemble a full battery. Figure S29 (Supporting Information) shows that NHVO is synthesized successfully using the hydrothermal method, and its XRD peaks correspond well with those of the DFT cards. The morphology of NHVO comprises short nanorods, which were characterized via SEM (Figure S30). **Figure** **6**a shows no significant differences in the cyclic voltammetry (CV) curves of different electrolytes, indicating negligible effect of PB molecules on the redox reaction. Additionally, the Zn//NHVO cell containing the PB‐0.02 electrolyte exhibits a high current density and highly reversible redox peaks, suggesting good electrochemical reaction activity. The charge‐discharge curves at 200 mA g^−1^ current density (Figure 6b) show an agreement between the voltage plateau and redox peak. The rate performances (Figure 6c) exhibit approximately the same capacities for 2 m and PB‐0.02 electrolytes, indicating that the PB content does not affect the kinetic performance of NHVO. Figure 6d shows the cycling performances of full batteries with different electrolytes at 200 mA g^−1^ current density. Compared to the 2 m pure electrolyte, the performance of the full cell containing the PB electrolyte is more stable. This is due to embedding of hydrated Zn^2+^ ions (Zn(H_2_O)_n_^2+^) of large radius (5.5 Å)^[^ ^30^ ^]^ in the NHVO cathode in a 2 m electrolyte environment leads to significant lattice distortions in the host material during the discharge process and results in structural collapse^[^ ^30^ ^]^ when the lattice stress exceeds its limit.^[^ ^31^ ^]^ This fragile structure of the NHVO results in a rapid decrease in the specific capacitance and a limited lifetime.When the NHVO cathode is operated in PB‐0.02, the results are different because unlike in the 2 m electrolyte, where the first solvated structure is composed of 5 water molecules, the number of water in the PB‐0.02 solvated clusters is reduced to 3.7. The PB molecules in the solvated structure have a strong attraction to water, resulting in fewer water molecules bound around the Zn^2+^ ions. That is, when hydrated Zn^2+^ enters the NHVO structure, it reduces the NHVO structure strain. Thus, this results in smaller size of guest species in NHVO crystals reducing the lattice distortion and ensuring better structural stability during long term cycling.^[^ ^32^ ^]^ Further, the cycling stability of PB‐containing electrolytes is better in the long cycle at 1 A g^−1^ current density than that of other electrolytes (Figure 6e), proving that the PB addition stabilizes the NHVO structure.

The working mechanism of the Zn//NHVO full cell is elucidated in Figure 6f. Further, the self‐discharge behaviors of the two cells were monitored to determine the side effects of battery mitigation during storage. Figure 6g and Figure S31 (Supporting Information) reveal that the Zn//NHVO cell with the PB electrolyte exhibits a higher capacity retention (64.6%) than that of 2 m electrolyte (58.5%) after resting for 48 h. Further, PB‐0.02 has a lower charge‐transfer resistance and higher conductivity, as demonstrated by the EIS test (Figure S32, Supporting Information). Further, the cycling performance of the PB‐0.02 electrolyte was evaluated by assembling a pouch cell (Figure 6h). The pouch battery using PB‐0.02 electrolyte has 60 stable cycles with an average specific capacity of 270.33 mA h g^−1^ at 0.5 A g^−1^, whereas 2 m pure electrolyte exhibits only 26 cycles with an average capacity of 230.58 mA h g^−1^, demonstrating that the PB electrolyte suppresses the side reactions of the battery.

## 3 Conclusion

This study demonstrated experimentally and through theoretical calculations that the addition of trace amounts of PB additives achieved a comprehensive regulatory effect. The PB additive breaks the original hydrogen‐bonding network by regulating the electrolyte environment and constructs a strong hydrogen‐bonding network of PB−H_2_O to inhibit free water displaying high activity. Simultaneously, the electrolyte containing PB additives construct a multi‐coordinated solvent dissolution structure, thereby reducing the side reactions. Moreover, PB adsorption at the zinc anode interface promotes uniform electric field distribution and reduces contact with water molecules, thereby promoting Zn growth along the (002) surface. A strong SEI layer of organic‐inorganic hybrid ZnF_2_‐ZnS was established on the Zn anode by reductive decomposition of the coordinated panthenol and OTF^−^, further separating water penetration and homogenize Zn^2+^ plating. Therefore, PB‐0.02 electrolytes impart excellent cycle life to zinc anode and reversibility exceeding 2300 h at current densities of 0.5 and 1 mA cm^−2^. The assembled Zn//NHVO full cell exhibited stable cycling performance and high capacity retention. Therefore, the integrated modulation strategy proposed in this study successfully demonstrated a simple method for effectively inhibiting the growth of zinc dendrites and stabilizing the anode interface.

## Conflict of Interest

The authors declare no conflict of interest.

## Supporting information

Supporting Information

## Data Availability

The data that support the findings of this study are available from the corresponding author upon reasonable request.
