# Supplementary material for: Panthenol Additives with Multiple Coordination Sites Induce Uniform Zinc Deposition and Inhibited Side Reactions for High Performance Aqueous Zinc Metal Battery
Source: Adv Sci (Weinh). 2024 Jul 21;11(35):2402074. doi: 10.1002/advs.202402074 (PMC11425255; doi:10.1002/advs.202402074)
Supplement: Supplementary file 1 — Supporting Information [file ADVS-11-2402074-s001.docx]

**Supporting Information**

**Panthenol Additives with Multiple Coordination Sites Induce Uniform Zinc Deposition and Inhibited Side Reactions for High Performance Aqueous Zinc Metal battery**

*Ping Luo, Gongtao Yu, Wenwei Zhang, Zhen Huang, Yipeng Wang, Dongyao Zhu, Feiyang Chao, Yuyua Wang, Wenhui Zhong, Zhaoyang Wang, Shijie Dong*, and Qinyou An**

P. Luo, G. Yu, Z. Huang, Y. Wang, D. Zhu, F. Chao, Y. Wang, W. Zhong, S. Dong

Hubei Engineering Laboratory of Automotive Lightweight Materials and Processing, Hubei Provincial Key Laboratory of Green Materials for Light Industry, School of Materials and Chemical Engineering, Hubei University of Technology, Wuhan 430068, P. R. China

P. Luo, S. Dong

Hubei Longzhong Laboratory, 441000, Xiang Yang, Hubei, P. R. China

W. Zhang, Q. An

State Key Laboratory of Advanced Technology for Materials Synthesis and Processing, Wuhan University of Technology, Wuhan, 430070, P. R. China

Z. Wang

School of Chemistry and Materials Science, Hubei Engineering University, XiaoGan, 432000, P. R. China

٭Corresponding Author

E-mail: [anqinyou86@whut.edu.cn](mailto:anqinyou86@whut.edu.cn) (Q.Y. An)

**Experimental Section**

*Preparation of the Electrolytes*

2 M of Zn(OTF)_2_ (Zn(CF_3_SO_3_)_2_, MACKLIN, 99%) was prepared by dissolving 2mmol Zn(OTF)_2_ (Zn(CF_3_SO_3_)_2_, MACKLIN, 99%) in 1 mL deionized water (DI). For the electrolyte adding PB, adding different amounts of panthenol (MACKLIN, 99%) powder to 2 M Zn (OTF)_2_ electrolyte, the ratio of water to PB substance is (1: *x*, *x*=0.005, 0.002, 0.005, 0.008, and 0.01). Unless otherwise indicated, the optimal PB ratio in this study was 1:0.02, expressed as PB-0.02. The electrolyte of the Zn// NH_4_V_4_O_10_ battery is 2 M Zn (OTF)_2_, PB-0.02 and PB-0.1 aqueous solutions.

*Preparation of the Electrodes*

NH_4_V_4_O_10_ powders were synthesized by a hydrothermal method. In detail, 585 mg of NH_4_VO_3_ (MACKLIN, 99%) was added into 35 ml DI water. 945.5 mg of H_2_C_2_O_4_·2H_2_O (MACKLIN, 99%) was then added into the NH_4_VO_3_ solution under magnetically stirring. The obtained solution was transferred to a 50 ml Teflon-lined autoclave and heated at 140 °C for 12 h. After cooling, the products were collected and washed with DI water, then dried at 70 °C overnight to obtain NH_4_V_4_O_10_ powders.

The cathode slurry was composed of 70% as-prepared active material, 20% conductive agent (super P, Timcal) and 10% polymer binder (PVDF, MACKLIN). Then the cathode was prepared by coating the slurry on the stainless foil and was dried at 80°C under vacuum for 12 h. The mass loading of the active materials is about 1.0-2.0 mg cm^-2^. The anode was served by Zn foil only.

*Fabrication of Zn//Zn symmetric cell*

Two pieces of Zn foils with a thickness of 200 μm were used as two electrodes for a symmetric cell. Two different electrolytes (2 M Zn(OTF)_2_ and PB-*x*, *x*=0.005, 0.02, 0.05, 0.08, and 0.1) each with 80 μL were added into the coin cell with a piece of glass fiber as a separator.

*Characterization*

The morphologies of Zn foil andes were observed by field emission scanning electron

microscopy (FESEM, ZEISS Gemini 300), operated at 2 kV and 10 mA. X-ray photoelectron spectroscopy (XPS) measurements were carried out through a Thermo Scientific K-Alpha using monochromatic Al Kα radiation. The crystal structure and material composition information were gathered by X-ray diffraction (XRD, Rigaku, MiniFlex600, Cu Kα, XPS, H nuclear magnetic resonance (NMR spectroscopy (Bruker 600MHz), and Fourier Transform Infrared Spectrometer (Thermo Scientific Nicolet iS20).

*Electrochemical Measurements*

All batteries were assembled in the air using CR2016 coin cells and the performances of Zn//Zn symmetric cells and Zn//NH_4_V_4_O_10_ full cells were collected by the Land CT3002A battery test system. The electrolyte used 2 M Zn(OTF)_2_ in water, and all the electrodes were cut into circular pieces with a diameter of 12 mm. The stability of the zinc anode was tested using Zn//Zn cells with Glass fiber separators. In addition, non-woven fibrous membranes were used to test the cycling life under high current density. Coulombic efficiency (CE) measurements and deposition/stripping curve were performed on Zn//Cu half-cells. Electrochemical impedance spectroscopy (EIS) was carried out using a Corrtest electrochemical workstation (Corrtest, Wuhan, China) within the frequency range from 100 kHz to 0.1 Hz. Linear polarization 2measurements were carried out using a three-electrode system with bare Zn as the working electrode, Pt plate as the counter electrode, and Ag//AgCl as the reference electrode. Tafel curve and potentiostatic polarization were all performed on the Corrtest electrochemical workstation with a three-electrode system (Zn foil as work electrode, Pt as counter electrode, and Ag//AgCl as reference electrode). Cyclic voltammetry (CV) of the Zn//NHVO cells was recorded at the voltage of 0.2 V−1.5 V. The deposition behavior of the Zn anode in different electrolytes at 10 mA cm-2 was in situ examined by digital optical microscopy (Bresser 52-01005).

*MD Simulations*

Molecular dynamics (MD) simulations: Quantum chemistry calculations were first performed to optimize molecular geometries of panthenol anion using the Gaussian 16 package [gaussian]^[1]^ at B3LYP/6-311+G(d) level of theory. The atomic partial charges on these solvent molecules were computed by fitting to the molecular electrostatic potential at atomic centers with the Møller-Plesset second-order perturbation method and the correlation-consistent polarized valence cc-pVTZ(-f) basis set. The atomistic force field parameters for all ions and molecules are described in AMBER format and are taken from previous work [amber-ff].^[2]^ The cross-interaction parameters between different atom types are obtained from the Lorentz-Berthelot combination rule. A reference modeling system consisting of 200 Zn(OTF)_2_ ion pairs and 5500 SPE water molecules was constructed, and a considerable amount of panthenol was introduced corresponding to its concentration of 10 mM in real experiments. Atomistic simulations of this modeling system were performed using GROMACS package with cubic periodic boundary conditions [gromacs].^[3]^ The equations for the motion of all atoms were integrated using a classic Verlet leapfrog integration algorithm with a time step of 1.0 fs. A cutoff radius of 1.6 nm was set for short-range van der Waals interactions and real-space electrostatic interactions. The particle-mesh Ewald (PME) summation method with an interpolation order of 5 and a Fourier grid spacing of 0.15 nm was employed to handle long-range electrostatic interactions in reciprocal space. All simulation systems were first energetically minimized using a steepest descent algorithm, and thereafter annealed gradually from 700 K to room temperature (300 K) within 10 ns. All annealed 3 simulation systems were equili rated in an isothermal-isobaric (NPT) ensemble for 0 ns of h sical time maintained using a os -Hoover thermostat and a Parrinello-Rahman barostat with time coupling constants of 0.4 and 0.2 ps, respectively, to control the temperature at 300 K and the pressure at 1 atm. Atomistic simulations were further performed in a canonical ensemble (NVT) for 50 ns, and simulation trajectories were recorded at an interval of 100 fs for further structural and dynamical analysis.

*DFT calculations*

All the first-principles calculations were carried out using Vienna Ab-initio Simulation Package (VASP).^[4]^ The valence orbitals were treated by the projected augmented wave (PAW) method.^[5]^ For the exchange-correlation energy, the Perdue-Burke-Ernzerhof (PBE) functional was used in the calculations.^[6]^ Considering the van der Waals (vdW) interactions,^[7]^ D3 method with Becke-Johnson damping function was used.^[8]^ The plane-wave cutoff energy was set to 500 eV. During the structure optimization, the convergence criterion was set to 10^-6^ eV/cell in energy and 0.02 eV/Å in force. The Γ-centered k-mesh of 2 × 2 × 2 was used in the optimization of molecular strucutres and the adsorption between Zn ion and molecular. And a 3 × 3 × 1 k-mesh was used in the adsorption between the Zn slab and molecular. VASPKIT was used in the representation of Highest Occupied Molecular Orbital (HOMO) and Lowest Unoccupied Molecular Orbital (LUMO).^[9]^ VESTA was used in the structure visualization.^[10]^

The adsorption energy between Zn slab and molecular is defined by Equation (1):

*E*_ad_(Zn+molecular) = *E*_Zn+molecular_ - *E*_Zn_ - *E*_molecular_ (1)

where, *E*_Zn_*_+_*_molecular_ is the energy of adsorption structure of Zn slab and adsorbed molecular, *E*_Zn_ is the energy of Zn slab and *E*_molecular_ is the energy of absorbed molecular.

The adsorption energy between molecular and Zn ion is defined by Equation (2):

*E*_ad_(molecular+ion) = *E*_molecular+ion_ - *E*_molecular_ - *E*_ion_ (2)

where, *E*_molecular+ion_ is the energy of the adsorption structure of the molecular and Zn ion, and *E*_ion_ is the energy of Zn ion.





**Figure S1.** Raman spectra of electrolytes with different PB additive content.


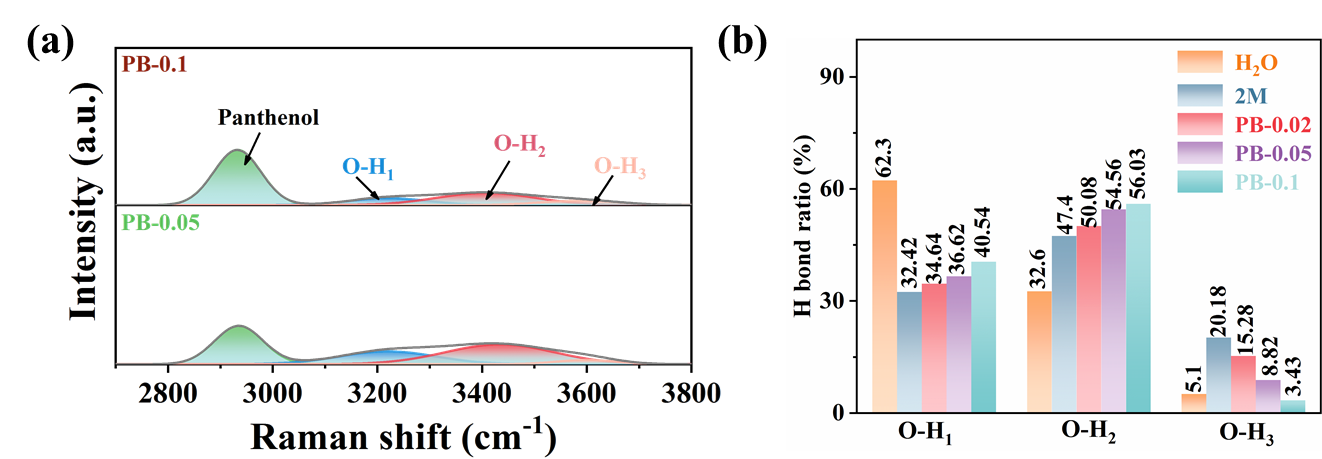


**Figure S2.** (a) Raman spectra of the PB-0.05, PB-0.1, (b) summary of the correlations of the H-bonds ratio.


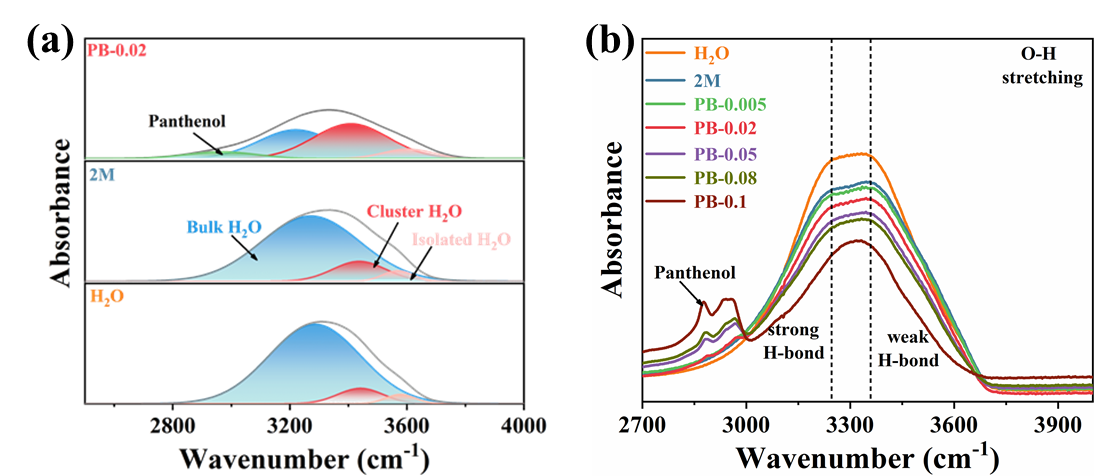


**Figure S3.** (a) FTIR spectra of H_2_O, 2M, and PB-0.02 electrolytes. (b) FTIR spectra of electrolytes with different PB additive content.


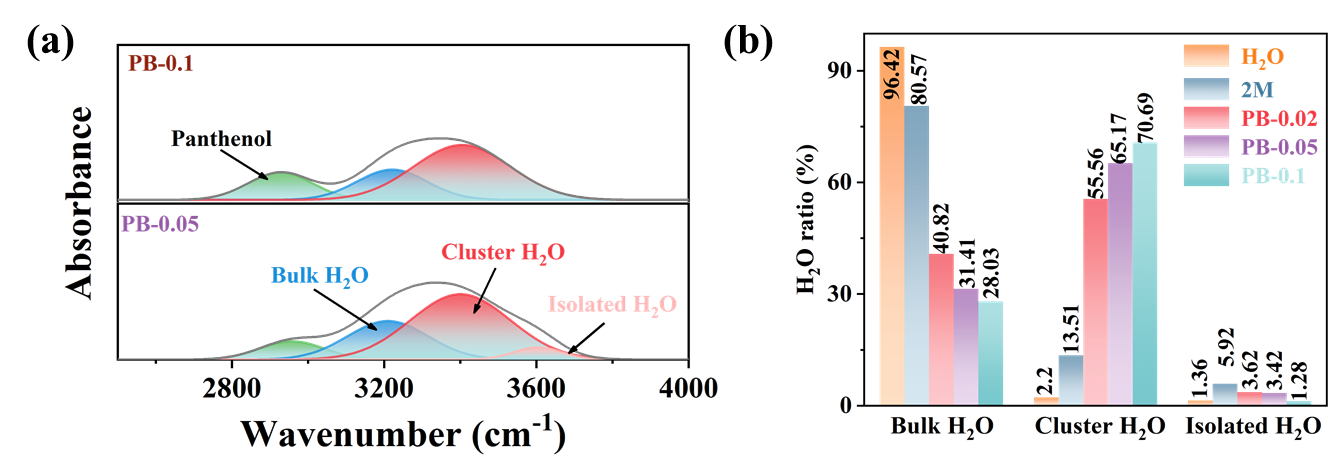


**Figure S4.** (a) FTIR spectra of the PB-0.05, PB-0.1, (b) summary of the correlations of the H_2_O ratio.





**Figure S5.** FTIR spectra of electrolytes with different PB additive content.


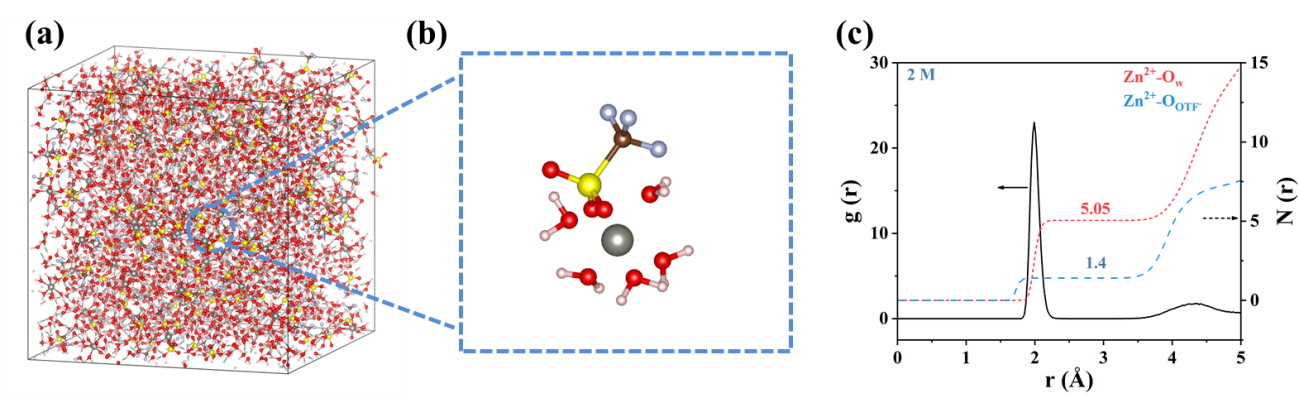


**Figure S6.** (a) 3D snapshot of MD simulations for 2M electrolyte and (b) partial enlarged snapshot representing Zn^2+^ solvation structure. (c) Simulated radial distribution functions (RDFs) for Zn^2+^−O_W_, Zn^2+^−O_OTF_^−^ collected from MD simulations in the 2M electrolyte.

**
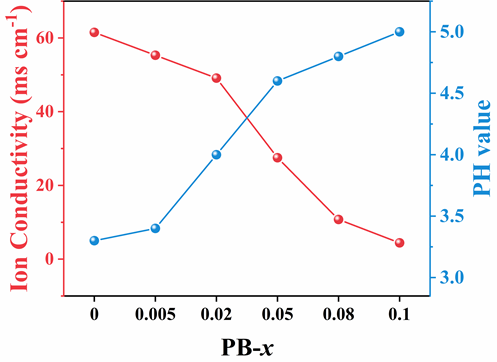
**

**Figure S7** Conductivity and PH value test of different proportion electrolyte.





**Figure S8.** Typical voltage-time curves of Zn//Cu asymmetric cells in different electrolytes.


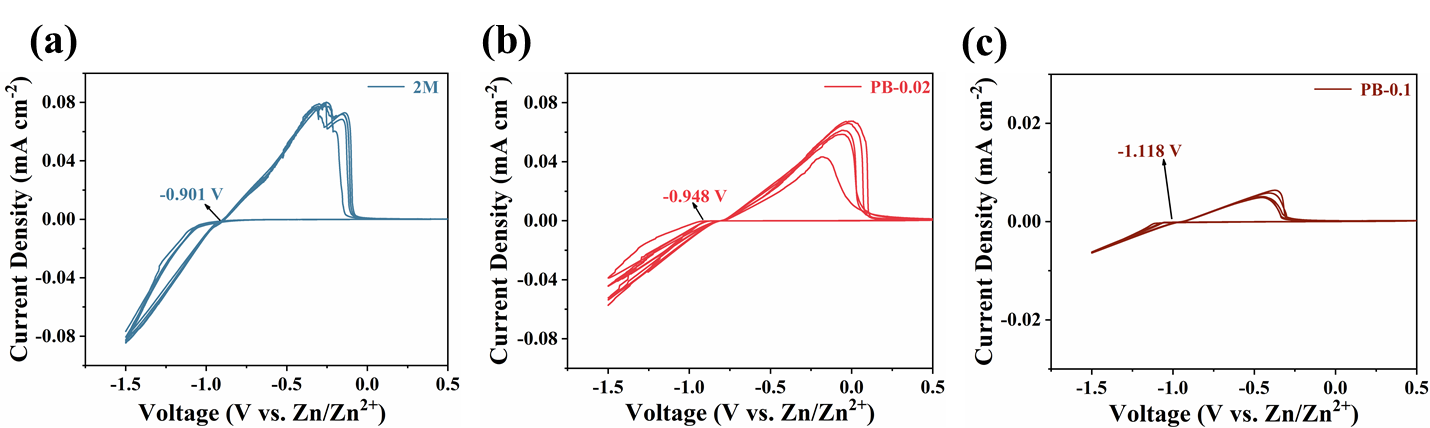


**Figure S9.** CV curves of Zn//Cu asymmetric cells in 2M, PB-0.02, and PB-0.1 electrolytes.

**

**

**Figure S10.** LSV testing of 2M electrolytes and PB-*x* hybrid electrolytes.

**

**

**Figure S11.** Tafel plots of Zn//Zn symmetric cells tested at a scan rate of 2 mV s^−1^ in different electrolytes.





**Figure S12.** EIS testing of different electrolytes.


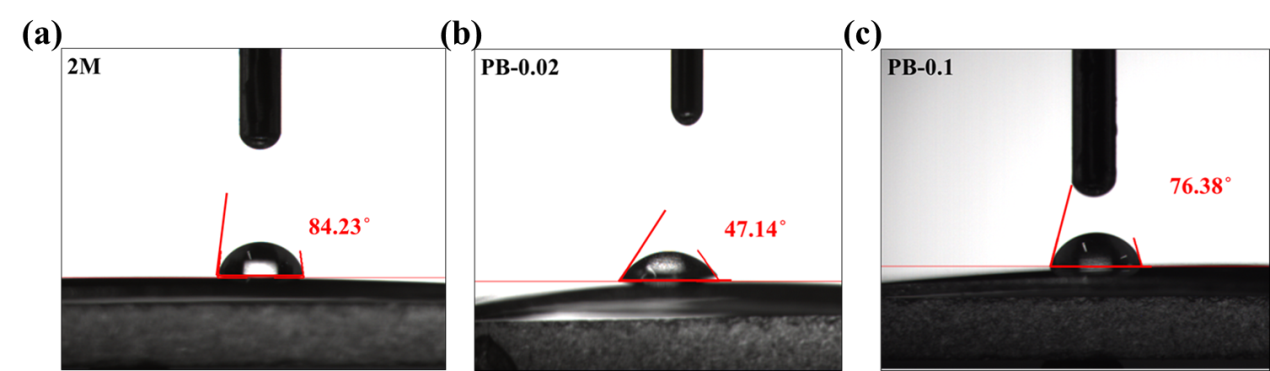


**Figure S13.** Contact angles of Zn anodes in 2M, PB-0.02, and PB-0.1 electrolytes.





**Figure S14.** Corresponding XRD patterns of Zn plates in different electrolytes.


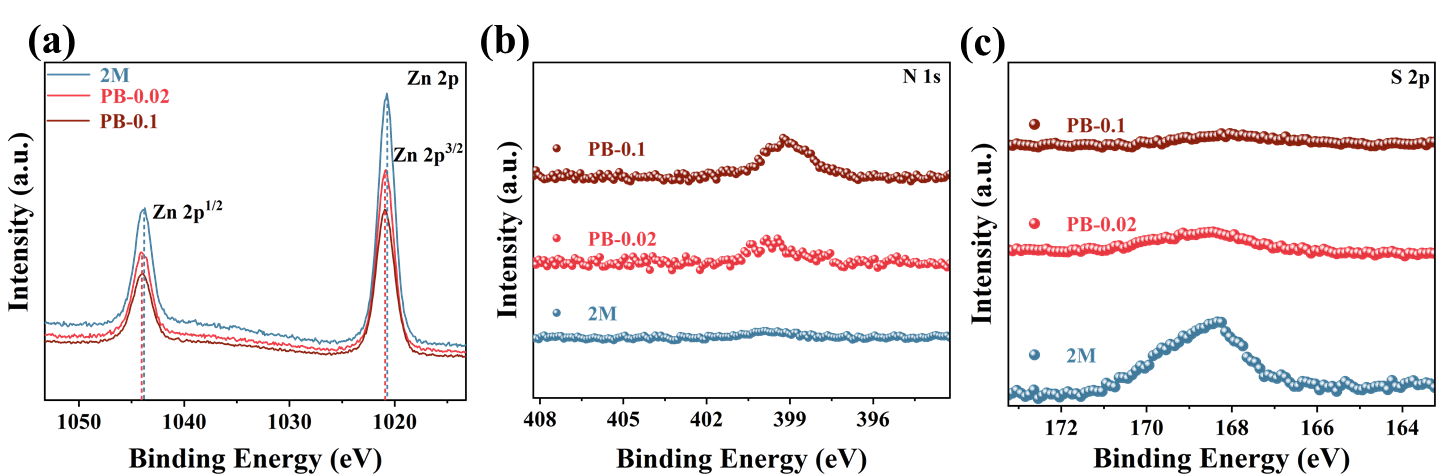


**Figure S15.** The anode surface XPS of different electrolytes were measured after 50 cycles at a current density of 1 mA cm^−2^ (a) Zn 2p, (b) N1s, and (c) S 2p.





**Figure S16.** Comparison of adsorption energies of H_2_O and PB molecule on the (100) plane of Zn anodes.


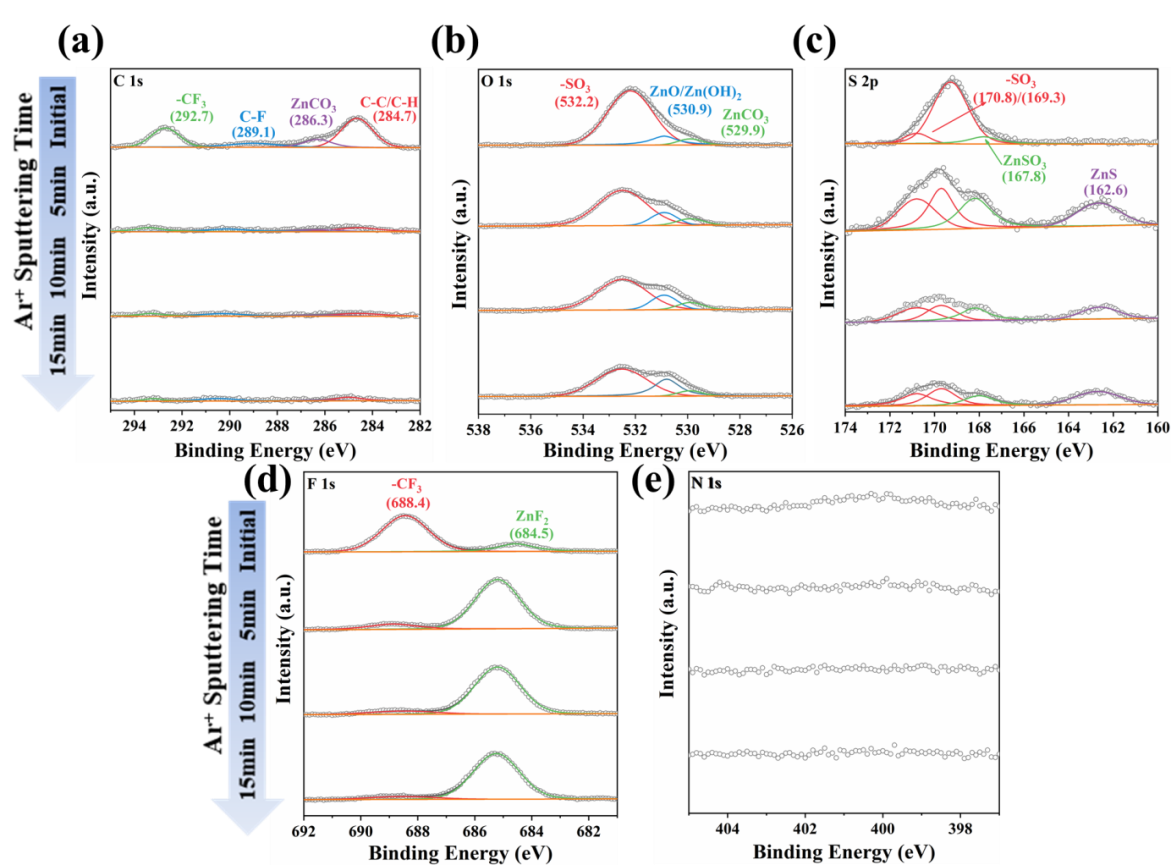


**Figure S17.** Time profiles of (a) C 1s, (b) O 1s, (c) S 2p, (d) F 1s, and (e) N 1s spectra generated after Ar^+^ sputtering for 0, 5, 10, and 15 min (Zinc anode after 50 cycles of 2M electrolyte).


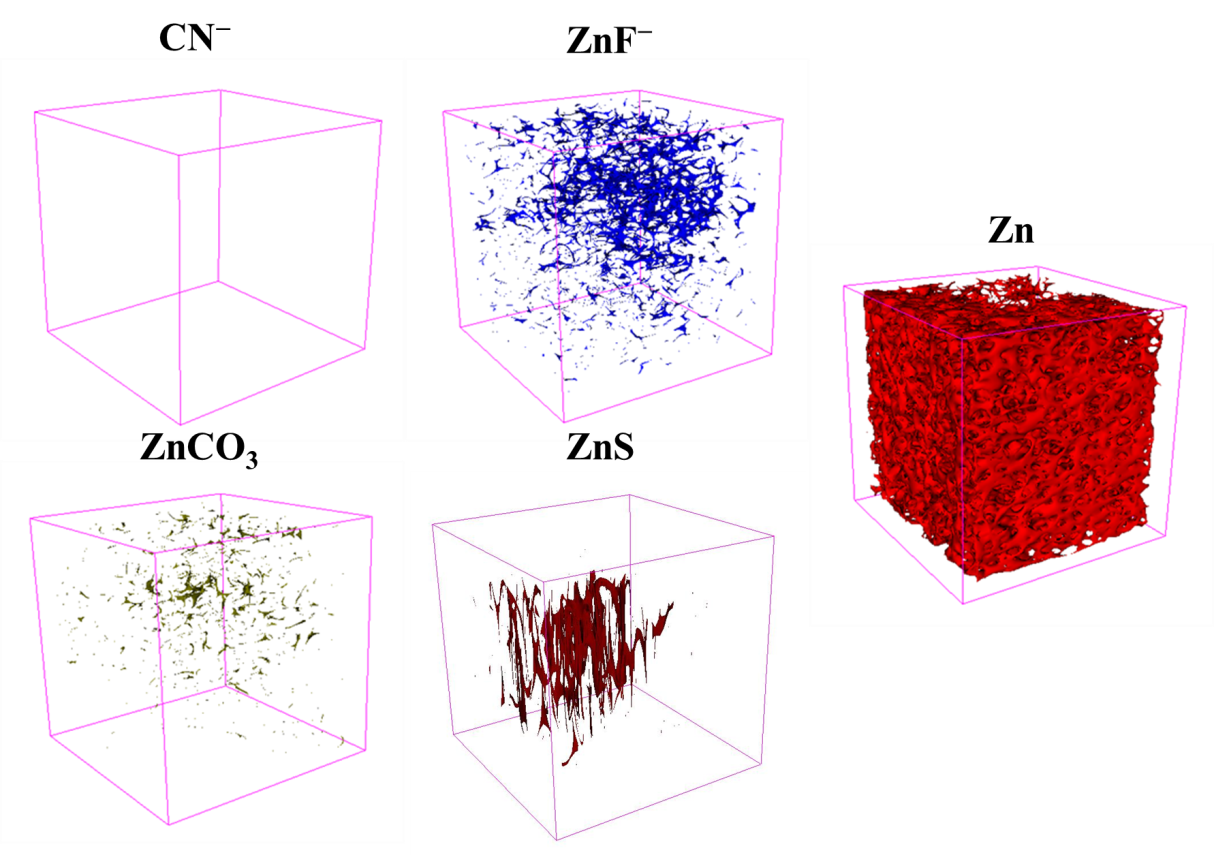


**Figure S18.** ToF-SIMS 3D rendering model of CN^−^, ZnF^−^, ZnCO_3_, ZnS, and Zn after 300s etching (Zinc anode after 50 cycles of 2M electrolyte).


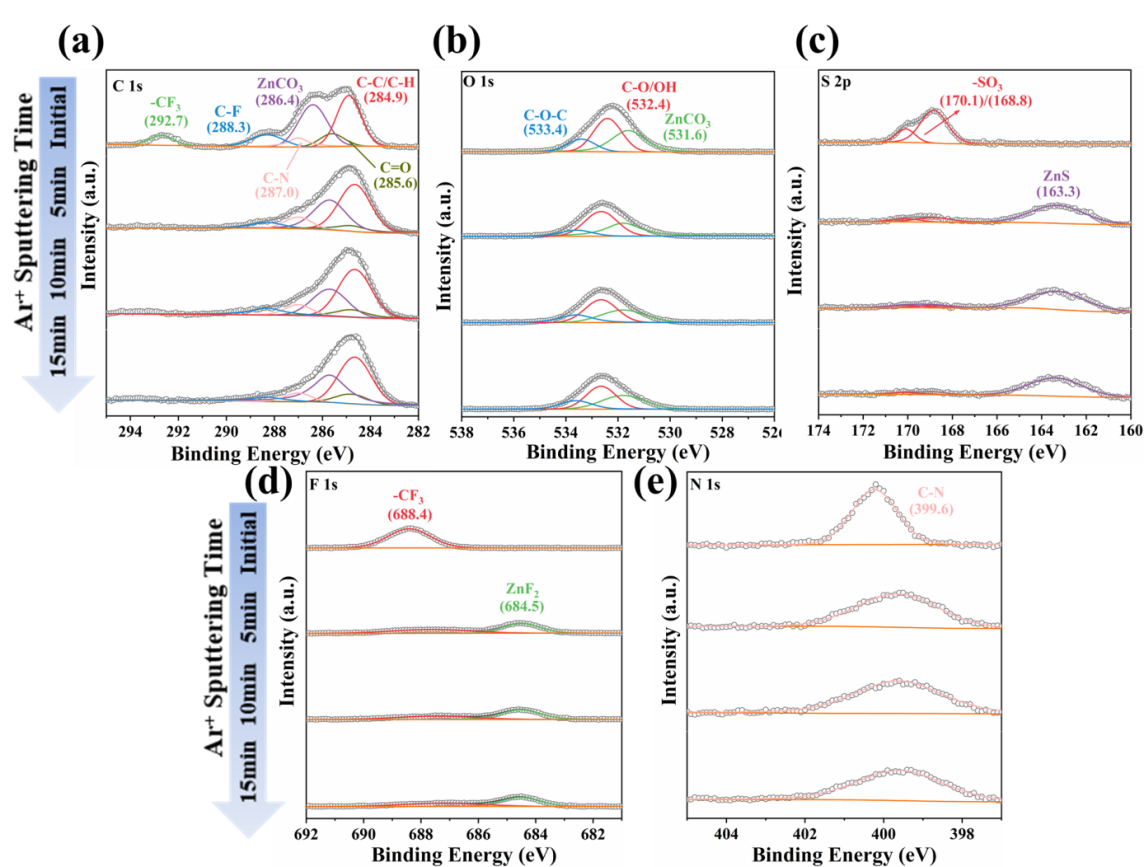


**Figure S19.** Time profiles of (a) C 1s, (b) O 1s, (c) S 2p, (d) F 1s, and (e) N 1s spectra generated after Ar^+^ sputtering for 0, 5, 10, and 15 min (Zinc anode after 50 cycles of PB-0.1 electrolyte).


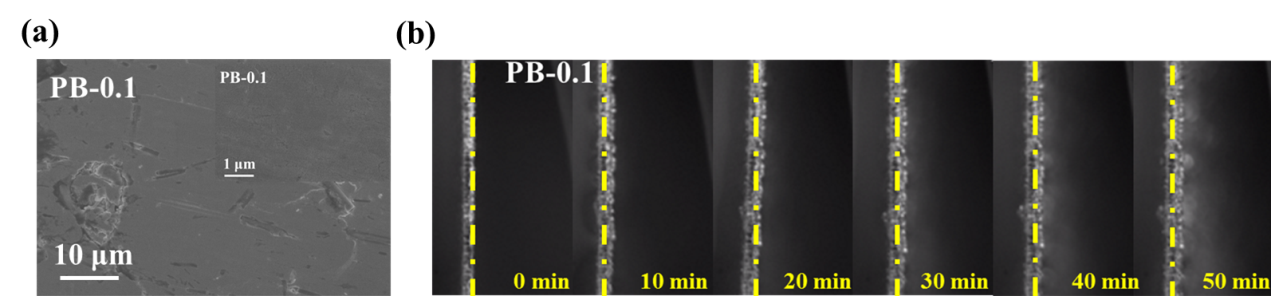


**Figure S20.** (a) SEM images of zinc anodes stripped from zinc//zinc symmetric cells after cycling at 1 mA cm^−2^,1 mA h cm^−2^ in the PB-0.1 electrolytes. (c) In *situ* optical microscope images of Zn plating in PB-0.01 electrolytes at a current density of 10 mA cm^−2^.


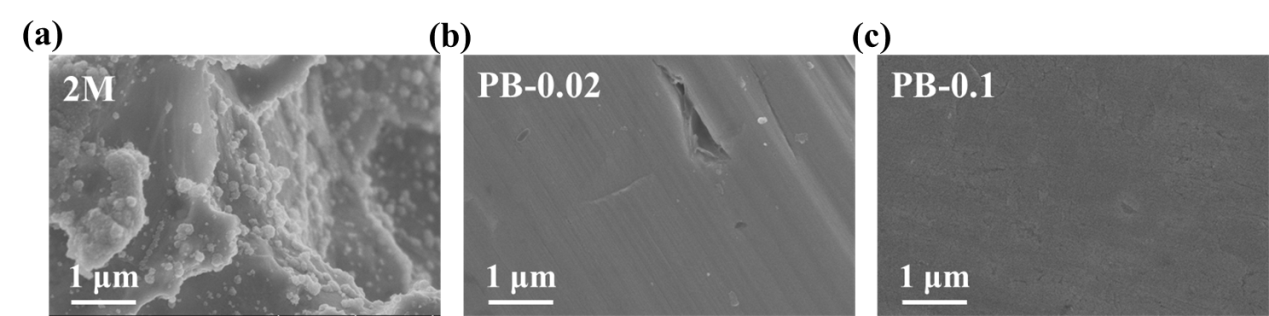


**Figure S21.** SEM images of zinc anodes stripped from zinc//zinc symmetric cells after cycling at 1 mA cm^−2^,1 mA h cm^−2^ in (a) 2M, (b) PB-0.02, and (c) PB-0.1 electrolytes.


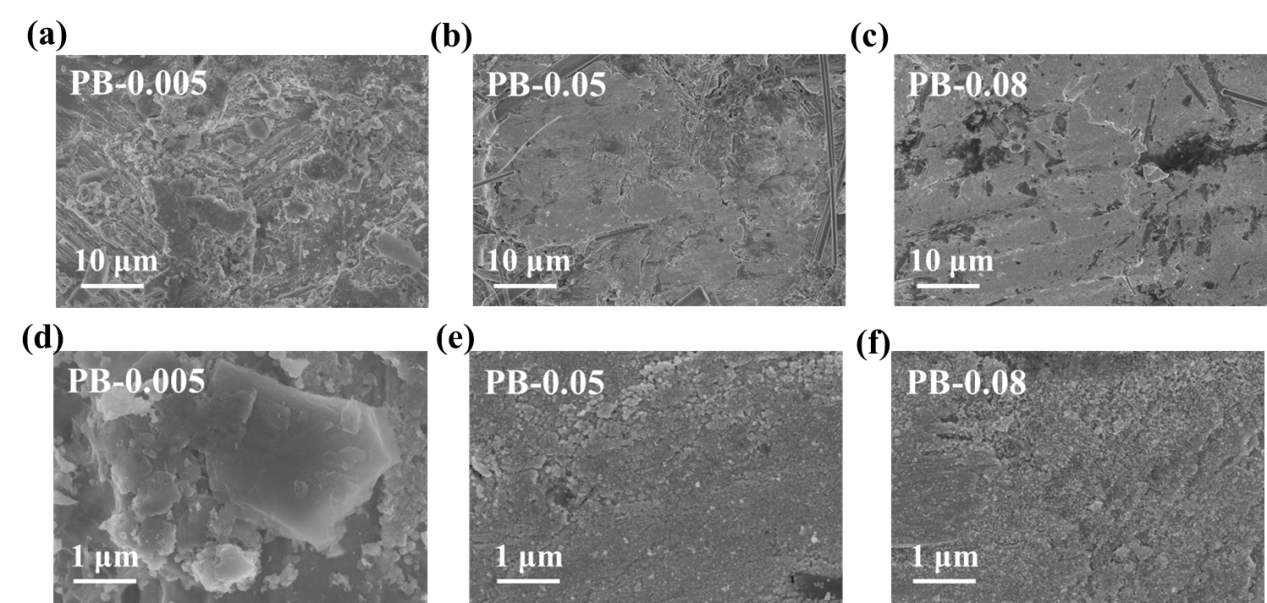


**Figure S22.** SEM images of zinc anodes stripped from zinc//zinc symmetric cells after cycling at 1 mA cm^−2^,1 mA h cm^−2^ in (a, c) PB-0.005, (b, e) PB-0.05, and (c, f) PB-0.08 electrolytes.


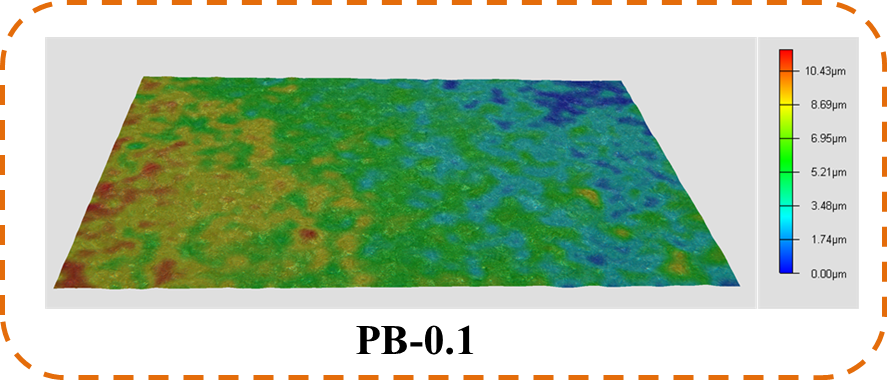


**Figure S23.** Laser confocal scanning microscopy images of the anode surface with different electrolytes after cycling at 1 mA cm^− 2^.


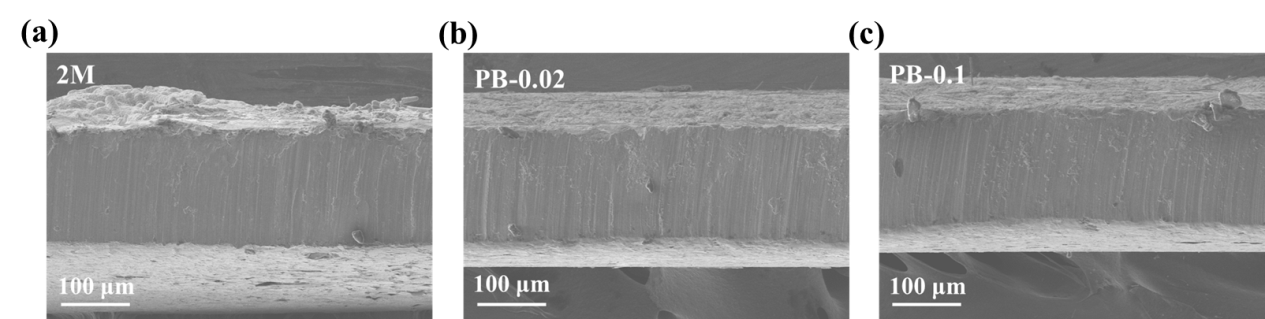


**Figure S24.** SEM images of zinc anodes section stripped from zinc//zinc symmetric cells after cycling at 1 mA cm^−2^,1 mA h cm^−2^ in (a) 2M, (b) PB-0.02, and (c) PB-0.1 electrolytes.


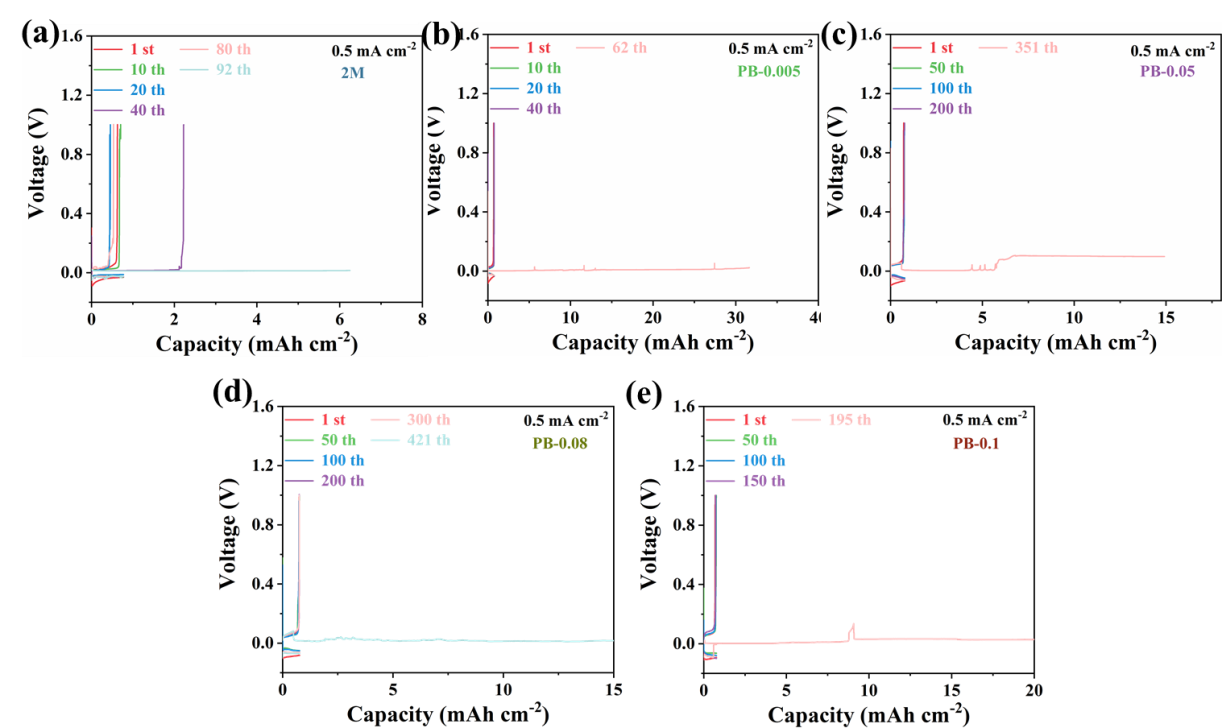


**Figure S25.** (a-e) Voltage profiles of Zn//Cu cells at different electrolytes.


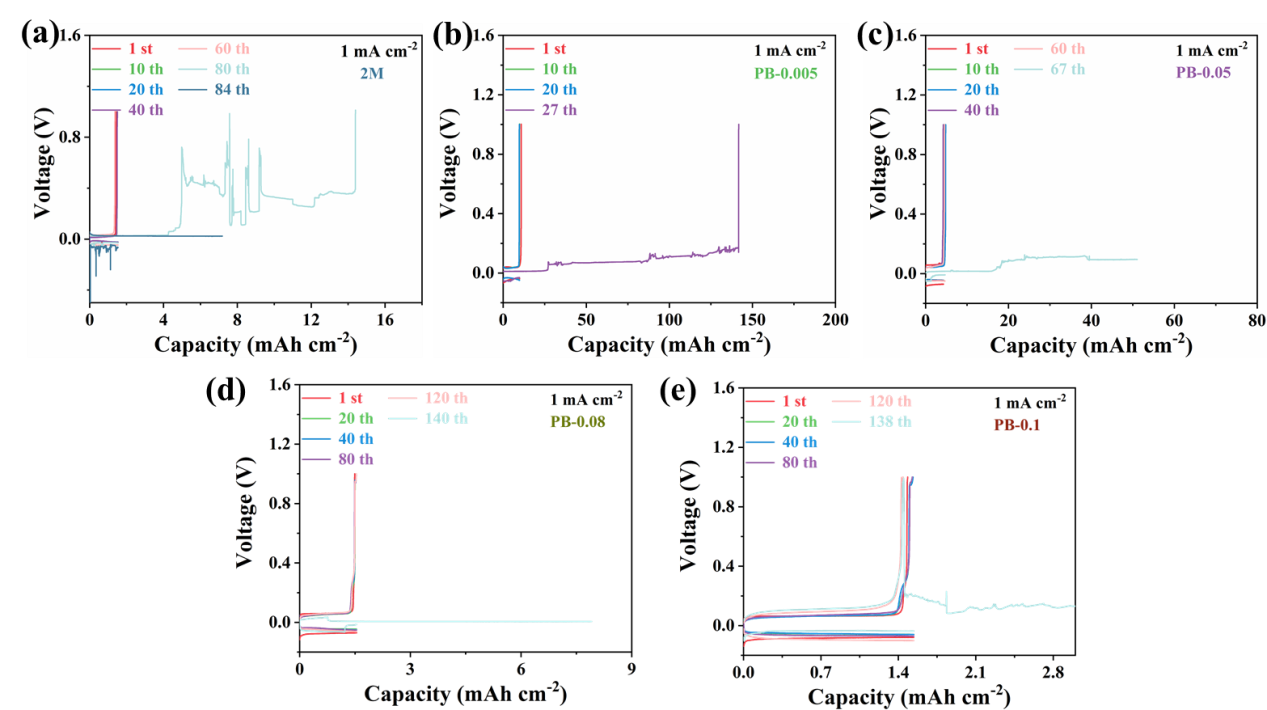


**Figure S26.** (a-e) Voltage profiles of Zn//Cu cells at different electrolytes.


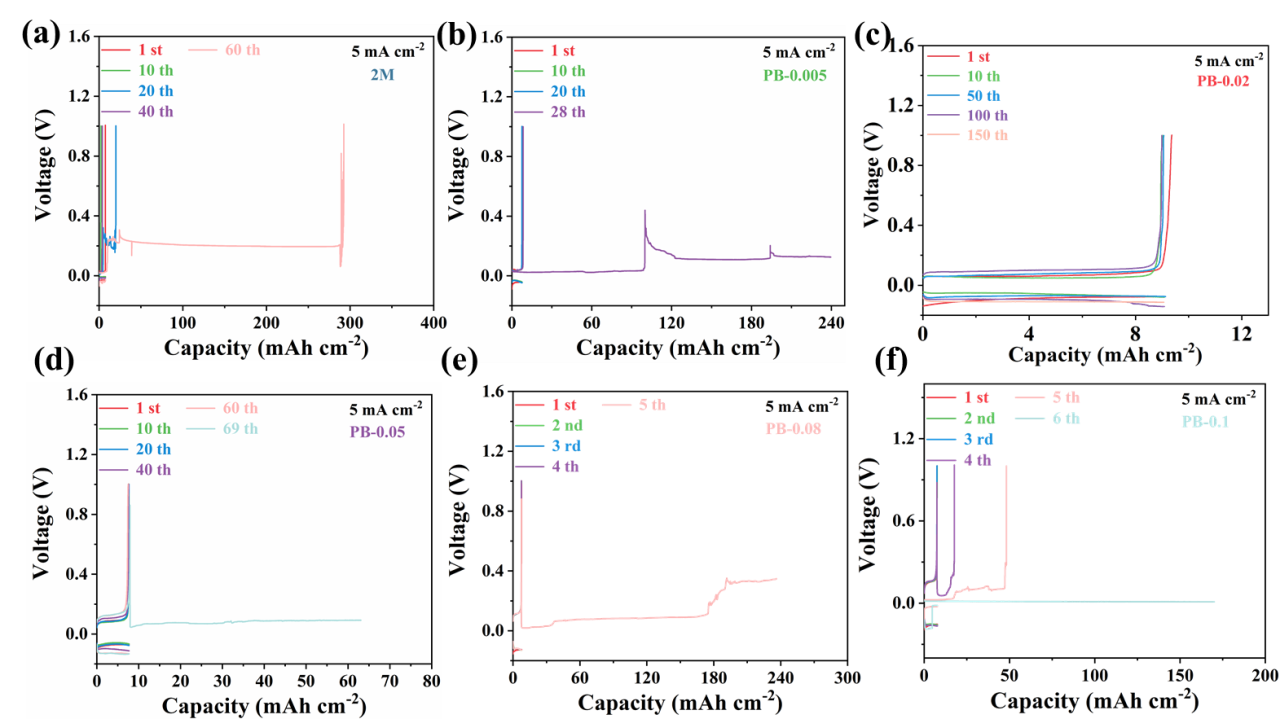


**Figure S27.** (a-f) Voltage profiles of Zn//Cu cells at different electrolytes.


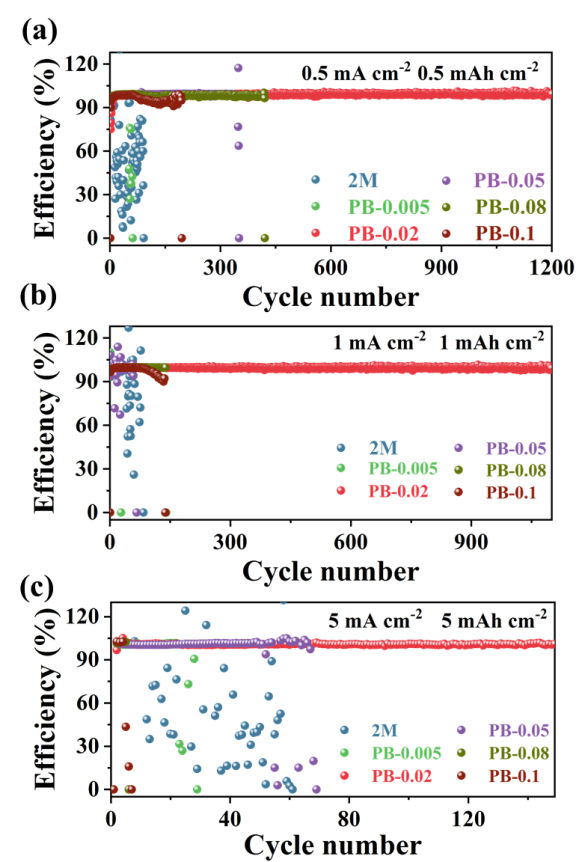


**Figure S28.** (a-c) CE performances of the asymmetrical Zn//Cu battery with difference electrolyte.





**Figure S29.** XRD pattern of NHVO.


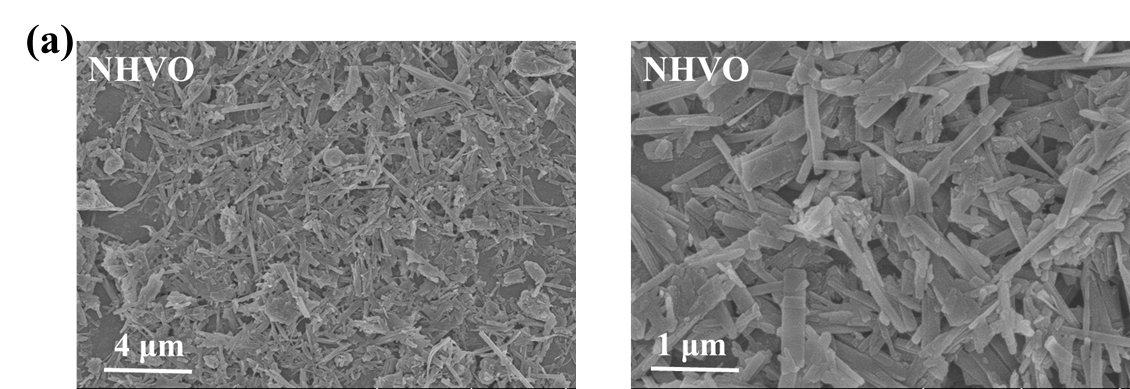


**Figure S30.** (a-b) SEM images of NHVO under different magnification microscope.





**Figure S31.** Self-discharge curves in 2M electrolytes.





**Figure S32.** EIS curves were measured in different electrolyte environments.

[1] M. e. Frisch, G. Trucks, H. B. Schlegel, G. Scuseria, M. Robb, J. Cheeseman, G. Scalmani, V. Barone, G. Petersson, H. Nakatsuji, Gaussian, Inc. Wallingford, CT, 2016.

[2] W. Yong-Lei, S. F. Ullah, G. Sergei, L. Aatto, **2014**.

[3] M. J. Abraham, T. Murtola, R. Schulz, S. Páll, J. C. Smith, B. Hess, E. Lindahl, *SoftwareX* **2015**, 1, 19.

[4] G. Kresse, J. Furthmüller, *Phys. Rev. B: Condens. Matter Mater. Phys.* **1996**, 54, 11169.

[5] Blöchl, P. E. Projector Augmented-Wave Method. *Phys. Rev. B* **1994**, 50, 17953.

[6] J. P. Perdew, K. Burke, M. Ernzerhof, *Phys. Rev. Lett.* **1996**, 77, 3865.

[7] S. Grimme, J. Antony, S. Ehrlich, S. Krieg, *J. Chem. Phys.* **2010**, 132, 154104.

[8] S. Grimme, S. Ehrlich, L. Goerigk, *J. Comput. Chem.* **2011**, 32, 1456.

[9] V. Wang, N. Xu, J. C. Liu, G. Tang, W. T. Geng, *Comput. Phys. Commun.* **2021**, 267, 108033.

[10] K. Momma, F. Izumi, *J. Appl. Crystallogr.* **2011**, 44, 1272.
